# Outcomes of In-Person and Telehealth Ambulatory Encounters During COVID-19 Within a Large Commercially Insured Cohort

**DOI:** 10.1001/jamanetworkopen.2022.8954

**Published:** 2022-04-26

**Authors:** Elham Hatef, Daniel Lans, Stephen Bandeian, Elyse C. Lasser, Jennifer Goldsack, Jonathan P. Weiner

**Affiliations:** 1 Division of General Internal Medicine, Department of Medicine, Johns Hopkins School of Medicine, Baltimore, Maryland; 2 Center for Population Health Information Technology, Department of Health Policy and Management, Johns Hopkins Bloomberg School of Public Health, Baltimore, Maryland; 3 Blue Health Intelligence, an independent licensee of the Blue Cross and Blue Shield Association, Chicago, Illinois; 4 Digital Medicine Society, Boston, Massachusetts

## Abstract

**Importance:**

Since the start of the COVID-19 pandemic, few studies have assessed the association of telehealth with outcomes of care, including patterns of health care use after the initial encounter.

**Objective:**

To assess the association of telehealth and in-person visits with outcomes of care during the COVID-19 pandemic.

**Design, Setting, and Participants:**

This cohort study assessed continuously enrolled members in private health plans of the Blue Cross and Blue Shield Association from July 1, 2019, to December 31, 2020.

**Main Outcomes and Measures:**

Main outcomes were ambulatory encounters per enrollee stratified by characteristics derived from enrollment files, practitioner claims, and community characteristics linked to the enrollee’s zip code. Outcomes of care were assessed 14 days after the initial encounters and included follow-up encounters of any kind, emergency department encounters, and hospitalizations after initial telehealth or in-person encounters.

**Results:**

In this cohort study of 40 739 915 individuals (mean [SD] age, 35.37 [18.77] years; 20 480 768 [50.3%] female), ambulatory encounters decreased by 1.0% and the number of in-person encounters per enrollee decreased by 17.0% from 2019 to 2020; however, as a proportion of all ambulatory encounters, telehealth encounters increased substantially from 0.6% (n = 236 220) to 14.1% (n = 5 743 718). For members with an initial telehealth encounter for a new acute condition, the adjusted odds ratio was 1.44 (95% CI, 1.42-1.46) for all follow-ups combined and 1.11 (95% CI, 1.06-1.16) for an emergency department encounter. For members with an initial telehealth encounter for a new chronic condition, the adjusted odds ratios were 0.94 (95% CI, 0.92-0.95) for all follow-ups combined and 0.94 (95% CI, 0.90-0.99) for in-patient admissions.

**Conclusions and Relevance:**

In this cohort study of 40.7 million commercially insured adults, telehealth accounted for a large share of ambulatory encounters at the peak of the pandemic and remained prevalent after infection rates subsided. Telehealth encounters for chronic conditions had similar rates of follow-up to in-person encounters for these conditions, whereas telehealth encounters for acute conditions seemed to be more likely than in-person encounters to require follow-up. These findings suggest a direction for future work and are relevant to policy makers, payers, and practitioners as they manage the use of telehealth during the COVID-19 pandemic and afterward.

## Key Points

**Question:**

What is the association of telehealth vs in-person encounters with outcomes of care during the COVID-19 pandemic in the US?

**Findings:**

In this cohort study of 40.7 million US commercially insured adults with acute clinical conditions, those with an initial telehealth encounter, compared with an in-person encounter, had higher odds for any follow-up encounter, an emergency department encounter, and in-patient admissions. For people with chronic conditions, the odds were lower for those with an initial telehealth encounter.

**Meaning:**

The contrasting patterns of follow-up care among members receiving telehealth for acute and chronic conditions have implications for health services during and after the COVID-19 pandemic.

## Teaser

This cohort study assesses outcomes associated with telehealth vs in-person ambulatory health care encounters among commercially insured US adults with acute clinical conditions during the COVID-19 pandemic.

## Introduction

Starting in 2020, the COVID-19 pandemic has resulted in notable changes in US health care. Much routine care was put on hold during the early months of the pandemic, whereas many hospitals were overwhelmed by patients seriously ill with COVID-19. At the same time, health insurance coverage of telehealth services was greatly expanded on an emergency basis to provide an alternative to in-person care without the risk of COVID-19 exposure. Thus, COVID-19 resulted in an unprecedented increase in the use of telehealth services during the early months of the pandemic.^1,2^

Since the surge of COVID-19, several studies^2,3,4,5,6,7,8,9,10,11,12,13,14^ have documented the expansion of telehealth services in the US, and few^15,16^ have assessed the association of telehealth with outcomes of care, including patterns of health care use after the initial encounter. This study expands on previous work.^2^ Here we assess the shifts in telehealth and ambulatory service use in the period after the initial implementation phase of telehealth. We document overall ambulatory encounters before and after the pandemic’s start within a large cohort of insured US patients. We also document key outcomes of care associated with an initial telehealth encounter compared with an in-person encounter. Finally, we assess the associations of the key patient, community, and health system characteristics with the likelihood of telehealth use.

## Methods

### Study Setting and Data Collection

The data set for this cohort study came from the repository of Blue Health Intelligence, a licensee of the Blue Cross and Blue Shield Association. The data set included claims files for commercial health plan members who used the commercial plan as their primary coverage. The study period was July 1, 2020, to December 31, 2020. eFigure 1 in the Supplement presents the sample selection and subgroup identification. We compared July to December 2020 with the same period in 2019 to account for seasonality. The study population was limited to members who were continuously enrolled from July 1, 2019, to December 31, 2020, and were covered through employer-based, Patient Protection and Affordable Care Act, and other private health insurance plans. The institutional review board of the Johns Hopkins Bloomberg School of Public Health approved this study as being exempt because it was a secondary analysis of de-identified data; therefore, patient informed consent was not required. This study conforms to the Strengthening the Reporting of Observational Studies in Epidemiology (STROBE) reporting guideline.^17^

Our unit of analysis was an ambulatory encounter with a telehealth-eligible service, excluding in-patient and emergency department (ED) services. We defined an encounter as a patient seeing a specific practitioner on a specific date and at a specific place of service. We identified telehealth-eligible services using the *Current Procedural Terminology* or *Healthcare Common Procedure Coding System* codes that, based on payer policy, were eligible for telehealth coverage,^18^ subdividing telehealth-eligible services between those provided in-person or via telehealth. eTables 1 and 2 in the Supplement present the list of telehealth-eligible services with the associated codes and designated codes for the place of service. We classified a telehealth-eligible service as provided via telehealth only when an appropriate modifier was present. We considered telehealth to include any synchronous service provided on a remote basis, whether via video or telephone. eTable 3 in the Supplement presents the approach to designating telehealth services and modality type. In the case of multiple claim lines per encounter (mean of 1.13), we selected the claim line with the highest allowed charge for each unique encounter. eTable 4 in the Supplement presents the study-designated specialty using practitioner specialty codes.

### Study Population

We captured information on the members’ demographic characteristics from enrollment files and mapped study members to the census region, state, county, and urban vs rural categorizations.^19^ Data on race and ethnicity were not available from the data repository. We documented the Elixhauser Comorbidity Index^20^ for each member based on all ambulatory care primary diagnoses noted during the 12 months of 2019.^21^

We calculated a rolling 7-day mean of new COVID-19 cases to compare different counties’ COVID-19 rates, using the data repository for the Johns Hopkins University COVID-19 Visual Dashboard,^22^ defining a COVID-19 hotspot of residence as any county where the rolling 7-day mean of COVID-19 cases was in the top decile throughout the country at any point during the study period. We assigned a member’s residence to 1 of 4 levels of social deprivation based on a national ranking of the Area Deprivation Index.^23^ We assessed internet connectivity using data from the 2019 US Census American Community Survey with a roll-up of census tract rates to zip code rates.^24^

We categorized the insurance type as standard preferred provider organization, high deductible (ie, >$1000), or health maintenance organization. Finally, we classified each encounter as involving a new patient if the enrollee had not visited the billing provider organization within the past 3 years and as a new condition if there had not been an encounter for that primary diagnosis within the past 3 years.^25^ All members of the cohort were continuously enrolled at least from July 2019, but to identify a new patient or a new condition we reviewed data from 2016 onward for those members who were enrolled longer.

### Outcome Measures

We assessed patterns of health care use 14 days after initial encounters, defining the use as follow-up encounters of any kind, ED encounters, or hospitalizations. We hypothesized that if a telehealth encounter was not as effective as an in-person encounter in addressing outstanding clinical issues, a higher rate of postindex follow-up encounters would be observed. We used the primary diagnosis reported for the encounter to characterize the clinical issues involved and used the list of ambulatory care sensitive (ACS)^26^ conditions,^21^ conditions that might result in an avoidable hospitalization if ambulatory care is inadequate in some respect.

We assessed the ambulatory encounters for each set of the 3-digit *International Statistical Classification of Diseases and Related Health Problems, Tenth Revision* (*ICD-10*) codes for ACS conditions. From the cohort of members who had 1 or more encounters during the study period, we counted the number of members who had an encounter for an ACS condition. To eliminate rare conditions, we limited the list to conditions that were diagnosed in at least 5000 members. To ensure adequate use of telehealth among the conditions, we required at least 10% of the members diagnosed with an ACS condition to have at least 1 telehealth claim associated with the condition. eTable 5 in the Supplement presents the approach to selecting acute and chronic ACS conditions for the assessment of use patterns.

We required members to have no condition-related preexisting care, defined as no encounters for the condition 90 days before the initial encounter. We categorized the ACS conditions to acute and chronic to account for the potential difference in telehealth and in-person encounters because of the nature of the conditions.

### Statistical Analysis

For selected ACS conditions, we used multivariate logistic regression to calculate odds ratios (ORs) and 95% CIs to assess the independent effect of the type of initial encounter on the likelihood of any 14-day follow-up encounters, adjusted for various member characteristics, including age, sex, Elixhauser Comorbidity Index based on 2019 ambulatory encounters, condition at the initial encounter, urban vs rural location of residence, member’s initial encounter coinciding in a COVID-19 hotspot, Area Deprivation Index, and internet connectivity levels. For chronic conditions, we included an encounter-level severity adjustment based on the primary diagnosis of the initial encounter. eTable 6 in the Supplement presents the assignment of a severity level to each complete *ICD-10* primary diagnosis within a 3-digit *ICD-10* category. This level was assigned based on clinical judgment as to the relative severity of the complete *ICD-10* code within the 3-digit category.

Only members with at least 1 telehealth-eligible service and at least 1 encounter in 2020 were included in the regression analysis (n = 1 086 720). The characteristics of this population are presented in eTable 7 in the Supplement. We performed a second logistic regression including an interaction term between the type of initial encounter and the specific ACS condition to address how telehealth effectiveness may vary by each clinical condition type. We used SAS, version 9.4 (SAS Institute Inc) to conduct the analyses.^27^

## Results

### Study Population

In this cohort study of 40 739 915 individuals (mean [SD] age, 35.37 [18.77] years; 20 480 768 [50.3%] female and 20 259 147 [49.7%] male), ambulatory encounters decreased by 1.0% and the number of in-person encounters per enrollee decreased by 17.0% from 2019 to 2020 (Table 1). As a proportion of all ambulatory encounters, telehealth encounters increased substantially from 0.6% (n = 236 220) to 14.1% (n = 5 743 718). eTable 8 in the Supplement presents the breakdown of the summer and fall trends, and eFigures 2 and 3 in the Supplement present weekly trends for total ambulatory encounters and the percentage delivered via telehealth in 2020.

**Table 1.** In-Person and Telehealth Ambulatory Encounters During the COVID-19 Pandemic for Continuously Enrolled Insured Members During July to December 2019 and 2020

Type of service	July to December	
	2019	2020
Total ambulatory encounters per enrollee, mean (SD) No.^a^	2.46 (5.00)	2.45 (5.24)
In-person ambulatory encounters per enrollee, mean (SD) No.^a^	2.45 (4.99)	2.04 (4.51)
Total encounters via telehealth, No. (%)	365 372 (0.4)	16 754 281 (16.8)
Video supported	126 316 (34.6)	13 350 610 (79.7)
Telephone	219 831 (60.2)	1 103 463 (6.6)
Other or unspecified	19 225 (5.3)	2 300 208 (13.7)
Enrollees with ≥1 ambulatory encounter of any kind, No. (%)	24 909 306 (61.1)	24 081 069 (59.1)
Enrollees with ≥1 telehealth encounter, No. (%)	236 220 (0.6)	5 743 718 (14.1)

^a^ The large SD compared with the mean is attributable to many members with 0 encounters for the year and to the skewed data (few members in the right tail of the distribution with a large number of encounters).

Table 2 presents the unadjusted member-level rates of total and telehealth ambulatory encounters according to member characteristics, and eTable 9 in the Supplement presents the breakdown of the summer and fall trends. In 2020, the highest telehealth uptake was seen in those aged 18 to 34 years (25.3% of per person encounters) and 35 to 49 years (19.0% of per person encounters), and more telehealth services were used in patients with a comorbidity index of 2 (19.6% of per person encounters) and 3 or higher (18.7% of per person encounters). Table 3 presents unadjusted encounter-level analyses stratified by encounter characteristics, and eTable 10 in the Supplement presents the breakdown of the summer and fall trends. The overall encounters decreased from a mean of 2.46 encounters per person in 2019 to 2.46 in 2020; however, for behavioral health encounters, the per person encounters increased from 0.32 in 2019 to 0.36 in 2020.

**Table 2.** Ambulatory Encounters and Percentage of Telehealth Encounters During July to December 2019 and 2020

Patient-level characteristic	Patients, No. (%)	2019 Mean (SD) No. of encounters per person^a^	2020	
			Mean (SD) No. of encounters per person^a^	Mean (SD) No. of encounters per person for those with ≥1 encounter (% telehealth)
Total	40 739 915	2.46 (5.00)	2.45 (5.24)	4.14 (16.80)
Age in 2019, y				
0-17	9 102 907 (22.3)	2.23 (6.01)	1.90 (6.25)	3.7 (19.22)
18-34	9 755 452 (24.0)	1.92 (4.19)	2.13 (4.62)	3.99 (25.27)
35-49	10 069 444 (24.7)	2.42 (4.57)	2.51 (4.83)	4.17 (19.04)
≥50	11 812 112 (29.0)	3.11 (5.04)	3.08 (5.14)	4.48 (12.57)
Sex				
Female	20 480 768 (50.3)	2.83 (5.10)	2.84 (5.40)	4.44 (19.48)
Male	20 259 147 (49.7)	2.09 (4.87)	2.05 (5.05)	3.78 (15.96)
Elixhauser Comorbidity Index				
0	38 294 865 (94.0)	2.25 (4.79)	2.30 (5.11)	4.01 (18.27)
1	2 354 975 (5.8)	5.57 (6.48)	4.67 (6.41)	5.4 (15.84)
2	69 213 (0.2)	10.43 (9.86)	7.08 (8.83)	7.86 (19.59)
≥3	20 862 (0.05)	13.04 (10.83)	8.71 (9.90)	9.42 (18.69)
US Census region of residence				
Midwest	10 056 251 (24.7)	2.49 (5.61)	2.50 (5.89)	4.35 (16.30)
Northeast	7 036 675 (17.23)	2.60 (5.01)	2.62 (5.39)	4.24 (25.44)
South	16 771 293 (41.2)	2.42 (4.57)	2.46 (4.77)	3.93 (13.99)
West	6 875 696 (16.9)	2.37 (5.05)	2.17 (5.18)	4.32 (22.84)
Urban or rural status of residence				
Rural	6 308 636 (15.5)	2.13 (3.89)	2.12 (4.04)	3.6 (10.61)
Urban	34 431 279 (84.5)	2.52 (5.18)	2.51 (5.43)	4.24 (19.16)
COVID-19 hotspot of residence				
No	18 676 928 (45.8)	2.63 (5.51)	2.63 (5.85)	4.44 (22.08)
Yes	22 062 987 (54.2)	2.32 (4.52)	2.29 (4.66)	3.89 (14.07)
Area Deprivation Index (by quartile)				
1 (Low deprivation)	16 869 885 (41.4)	2.74 (5.68)	2.70 (5.98)	4.5 (21.85)
2	9 163 847 (22.5)	2.35 (4.65)	2.33 (4.86)	3.97 (15.91)
3	8 582 511 (21.1)	2.27 (4.40)	2.27 (4.59)	3.86 (14.11)
4 (High deprivation)	6 123 672 (15.0)	2.12 (4.20)	2.18 (4.40)	3.78 (13.95)
No. of households with internet access per 1000 at zip code level				
0-499	164 834 (0.4)	1.99 (3.65)	2.05 (3.79)	3.54 (2.12/8.13^b^)
500-1000	40 575 081 (99.6)	2.46 (5.01)	2.45 (5.25)	4.14 (1.11/13.38^b^)
Type of insurance plan in 2019				
High deductible	10 623 412 (26.1)	2.14 (4.30)	2.15 (4.46)	3.72 (15.09)
HMO	2 435 534 (6.0)	2.43 (4.68)	2.14 (4.99)	4.37 (28.90)
Standard PPO	27 680 969 (68.0)	2.59 (5.27)	2.59 (5.53)	4.28 (18.15)

Abbreviations: HMO, health maintenance organization; PPO, preferred provider organization.

^a^ The entire sample (of users and nonusers) continuously enrolled in commercial insurance plans from July 1, 2019, through December 31, 2020. These unadjusted rates are reported as the number of telehealth-eligible ambulatory encounters per enrollee and the percentage of these encounters that took place via telehealth during the study period (July to December). All rows are calculated at the person level.

^b^ Telephone/video encounters.

**Table 3.** Ambulatory Encounters and Percentage of Telehealth Encounters During July to December 2019 and 2020

Encounter-level characteristic	2019 Mean (SD) No. of encounters per person^a^	2020	
		Mean (SD) No. of encounters per person^a^	Mean No. of encounters per person for those with ≥1 encounter (% telehealth)
Total	2.46 (5.00)	2.45 (5.24)	4.14 (16.80)
Type of ambulatory encounter			
Evaluation and management office	1.60 (2.39)	1.56 (2.43)	2.64 (14.61)
Behavioral health	0.32 (2.50)	0.36 (2.53)	0.6 (53.41)
Rehabilitation	0.40 (2.53)	0.38 (2.83)	0.64 (1.66)
Other	0.14 (1.04)	0.15 (1.10)	0.26 (10.40)
Practitioner specialty			
Primary care (MD or DO)	0.69 (1.31)	0.62 (1.25)	1.06 (15.23)
Medical specialist (MD or DO)	0.22 (0.75)	0.23 (0.80)	0.39 (13.58)
Surgical specialist (MD or DO)	0.20 (0.77)	0.20 (0.76)	0.33 (3.64)
Behavioral health (MD, DO, or non-MD)	0.17 (1.66)	0.18 (1.87)	0.31 (58.06)
Rehabilitation (MD, DO, or non-MD)	0.33 (2.27)	0.32 (2.26)	0.53 (2.74)
Other physician	0.52 (2.42)	0.56 (2.65)	0.95 (24.85)
Physician assistant or nurse practitioner	0.19 (0.70)	0.21 (0.75)	0.35 (13.65)
Other nonphysicians	0.13 (1.20)	0.13 (1.27)	0.21 (18.58)
Primary diagnosis			
Diabetes	0.05 (0.37)	0.05 (0.37)	0.09 (13.85)
Hypertension	0.07 (0.34)	0.07 (0.36)	0.12 (12.28)
Behavioral health	0.46 (3.04)	0.50 (3.35)	0.85 (51.44)
Cancer	0.05 (0.50)	0.05 (0.55)	0.09 (8.07)
Well-child care	0.02 (0.16)	0.02 (0.14)	0.03 (0.30)
COVID-19 diagnosis	NA	0.02 (0.20)	0.04 (27.25)
Other chronic diagnosis	0.44 (1.46)	0.42 (1.46)	0.71 (12.66)
Other acute diagnosis	3.04 (1.38)	1.31 (3.04)	2.22 (7.93)
Continuity of patient encounter or problem			
Existing patient encounters	1.90 (4.68)	1.81 (4.80)	3.06 (18.07)
New patient encounters	0.56 (2.29)	0.64 (2.61)	1.08 (17.84)
Existing problem encounters	1.58 (4.54)	1.49 (4.60)	2.53 (18.76)
New problem encounters	0.88 (2.67)	0.95 (3.03)	1.61 (16.85)

Abbreviations: DO, doctor of osteopathic medicine; MD, doctor of medicine; NA, not applicable.

^a^ The full study sample included members continuously enrolled from July 1, 2019, through December 31, 2020. These unadjusted rates are reported as the number of telehealth-eligible ambulatory encounters per enrollee that took place during the study period (July to December). Percentages represent the proportion of encounters that took place via telehealth. Note that all rows were calculated at the encounter level and that all the per persons columns include all enrollees whether or not they used any services.

### Association With Outcomes of Care

Table 4 presents the results of the logistic regression model. In the cohort with acute ACS conditions, the adjusted ORs for those with an initial telehealth encounter, compared with in-person, were 1.44 (95% CI, 1.42-1.46) for a follow-up encounter of any kind and 1.11 (95% CI, 1.06-1.16) for an ED encounter. eTable 11 in the Supplement presents a summary of ACS conditions and subsequent use patterns during 2020. Among the cohort with chronic ACS conditions, the adjusted ORs were 0.94 (95% CI, 0.92-0.95) for follow-up encounters of any kind and 0.94 (95% CI, 0.90-0.99) for hospitalization. Table 5 presents the patterns of subsequent use among those with acute and chronic ACS conditions by the clinical condition. For instance, among those having an acute upper respiratory tract infection episode, the ORs for members with an initial telehealth encounter, compared with in-person encounters, were 1.65 (95% CI, 1.61-1.68) for a follow-up encounter of any kind, 1.18 (95% CI, 1.10-1.25) for an ED follow-up encounter, and 1.11 (95% CI, 1.03-1.20) for hospitalization. Among patients with a chronic ACS condition, the ORs were 0.86 (95% CI, 0.84-0.88) for members with essential hypertension and 0.63 (95% CI, 0.50-0.80) for members with heart failure who had initial telehealth encounters, compared to in-person encounters, for a follow-up encounter of any kind.

**Table 4.** Association of Telehealth and Other Key Factors With Likelihood of Any, Emergency, or Hospitalization Follow-ups for Patients With Acute and Chronic Ambulatory Care Sensitive Conditions

Key independent variable	Patients, No. (%)	Adjusted OR^a^ (95% CI)		
		Any follow-up encounter	ED follow-up	Hospitalization follow-up
**Acute ambulatory care sensitive conditions**				
First encounter				
Telehealth	113 857 (18.7)	1.44 (1.42-1.46)	1.11 (1.06-1.16)	1.03 (0.98-1.08)
In person	493 716 (81.3)	1 [Reference]	1 [Reference]	1 [Reference]
Age in 2019, y				
0-17	186 295 (30.7)	0.38 (0.38-0.39)	0.49 (0.46-0.52)	0.22 (0.20-0.23)
18-34	166 488 (27.4)	0.61 (0.60-0.62)	0.98 (0.93-1.03)	0.51 (0.48-0.54)
35-49	133 412 (22.0)	0.78 (0.76-0.79)	1.03 (0.99-1.08)	0.60 (0.57-0.63)
≥50	121 378 (20.0)	1 [Reference]	1 [Reference]	1 [Reference]
Sex				
Female	350 067 (57.6)	1.43 (1.42-1.45)	1.27 (1.22-1.31)	1.04 (1.00-1.09)
Male	257 506 (42.4)	1 [Reference]	1 [Reference]	1 [Reference]
Elixhauser Comorbidity Index				
0	571 199 (94.0)	1 [Reference]	1 [Reference]	1 [Reference]
1	35 022 (5.8)	1.90 (1.85-1.96)	1.72 (1.62-1.81)	1.80 (1.70-1.90)
2	985 (0.2)	2.87 (2.39-3.45)	1.98 (1.50-2.62)	2.85 (2.22-3.65)
≥3	367 (0.1)	4.25 (2.97-6.07)	2.55 (1.71-3.82)	3.09 (2.10-4.53)
Urban or rural status of residence				
Rural	127 547 (21.0)	0.83 (0.82-0.85)	0.95 (0.91-1.00)	0.89 (0.84-0.93)
Urban	480 026 (79.0)	1 [Reference]	1 [Reference]	1 [Reference]
COVID-19 hotspot residence^b^				
Hotspot (top decile)	46 646 (7.7)	1.12 (1.10-1.14)	1.37 (1.30-1.45)	1.29 (1.20-1.37)
Not a hotspot	560 927 (92.3)	1 [Reference]	1 [Reference]	1 [Reference]
Area Deprivation Index (by quartile)				
1	218 990 (36.0)	1 [Reference]	1 [Reference]	1 [Reference]
2	147 410 (24.3)	0.87 (0.85-0.88)	1.25 (1.20-1.31)	1.06 (1.00-1.12)
3	143 458 (23.6)	0.85 (0.84-0.87)	1.34 (1.28-1.40)	1.14 (1.08-1.20)
4	97 715 (16.1)	0.85 (0.84-0.87)	1.36 (1.29-1.44)	1.21 (1.14-1.29)
**Chronic ambulatory care sensitive conditions**				
First encounter				
Telehealth	94 481 (18.7)	0.94 (0.92-0.95)	0.96 (0.92-1.01)	0.94 (0.90-0.99)
In person	410 743 (81.3)	1 [Reference]	1 [Reference]	1 [Reference]
Age in 2019, y				
0-17	33 450 (6.6)	0.51 (0.49-0.52)	0.72 (0.65-0.79)	0.34 (0.30-0.38)
18-34	67 634 (13.4)	0.75 (0.73-0.76)	1.27 (1.20-1.35)	0.71 (0.66-0.75)
35-49	146 240 (29.0)	0.88 (0.87-0.89)	1.20 (1.15-1.26)	0.74 (0.71-0.77)
≥50	257 900 (51.1)	1 [Reference]	1 [Reference]	1 [Reference]
Sex				
Female	245 925 (48.7)	1.53 (1.51-1.55)	1.40 (1.35-1.46)	1.07 (1.03-1.11)
Male	259 299 (51.3)	1 [Reference]	1 [Reference]	1 [Reference]
Elixhauser Comorbidity Index				
0	422 483 (83.6)	1 [Reference]	1 [Reference]	1 [Reference]
1	80 052 (15.8)	1.40 (1.38-1.43)	1.46 (1.39-1.54)	1.42 (1.36-1.49)
2	1993 (0.4)	2.77 (2.43-3.15)	2.01 (1.59-2.55)	2.12 (1.75-2.58)
≥3	696 (0.1)	2.90 (2.31-3.65)	2.65 (1.89-3.72)	3.55 (2.73-4.61)
Severity index (for primary diagnosis of the index encounter)				
1	463 544 (91.8)	1 [Reference]	1 [Reference]	1 [Reference]
2	40 032 (7.9)	1.05 (1.02-1.07)	1.12 (1.04-1.21)	1.19 (1.10-1.28)
3	1648 (0.3)	1.19 (1.06-1.33)	0.98 (0.72-1.32)	1.50 (1.20-1.88)
Urban or rural status of residence				
Rural	89 174 (17.6)	0.87 (0.86-0.89)	1.00 (0.95-1.05)	0.84 (0.80-0.89)
Urban	416 050 (82.4)	1 [Reference]	1 [Reference]	1 [Reference]
COVID-19 hotspot residence^b^				
Hotspot (top decile)	33 816 (6.7)	1.16 (1.13-1.19)	1.51 (1.42-1.62)	1.26 (1.18-1.35)
Not a hotspot	471 408 (93.3)	1 [Reference]	1 [Reference]	1 [Reference]
Area Deprivation Index (by quartile)				
1	175 821 (34.8)	1 [Reference]	1 [Reference]	1 [Reference]
2	115 013 (22.8)	0.91 (0.89-0.92)	1.28 (1.21-1.36)	1.05 (0.99-1.10)
3	120 159 (23.8)	0.87 (0.86-0.89)	1.43 (1.35-1.51)	1.10 (1.04-1.15)
4	94 231 (18.6)	0.83 (0.82-0.85)	1.48 (1.40-1.57)	1.18 (1.12-1.24)

Abbreviations: ED, emergency department; OR, odds ratio.

^a^ The models are adjusted for the type of acute and chronic ambulatory care sensitive conditions treated during the episode. See Table 5 for a list of conditions in the acute and chronic categories. See eTable 5 in the Supplement for more information on the approach to selecting acute and chronic ambulatory care sensitive conditions.

^b^ A member was identified as being in a COVID-19 hotspot if at least 1 of their encounters coincided with a COVID-19 hotspot during the entire study period.

**Table 5.** Association of Telehealth With Likelihood of Any, Emergency, or Hospitalization Follow-ups for Patients With Specific Acute and Chronic Ambulatory Care Sensitive Conditions^a^

Clinical condition	Patients, No. (%)	Adjusted OR (95% CI)		
		Any follow-up encounter	ED follow-up	Hospitalization follow-up
**Acute ambulatory care sensitive conditions**				
Acute bronchitis				
First encounter: telehealth	9245 (23.3)	1.23 (1.17-1.30)	1.18 (1.05-1.33)	1.04 (0.91-1.18)
First encounter: in person	30 381 (76.7)	1 [Reference]	1 [Reference]	1 [Reference]
Acute pharyngitis				
First encounter: telehealth	36 399 (16.1)	1.46 (1.42-1.49)	1.10 (1.01-1.19)	0.98 (0.89-1.08)
First encounter: in person	189 567 (83.9)	1 [Reference]	1 [Reference]	1 [Reference]
Acute pyelonephritis				
First encounter: telehealth	389 (19.0)	0.94 (0.71-1.26)	100 (0.65-1.53)	0.93 (0.56-1.54)
First encounter: in person	1655 (81.0)	1 [Reference]	1 [Reference]	1 [Reference]
Acute tonsillitis				
First encounter: telehealth	3758 (13.8)	1.03 (0.96-1.11)	0.80 (0.64-0.99)	0.86 (0.62-1.21)
First encounter: in person	23 424 (86.2)	1 [Reference]	1 [Reference]	1 [Reference]
Acute upper respiratory tract infections of multiple and unspecified sites				
First encounter: telehealth	49 336 (21.5)	1.65 (1.61-1.68)	1.18 (1.10-1.25)	1.11 (1.03-1.20)
First encounter: in person	180 034 (78.5)	1 [Reference]	1 [Reference]	1 [Reference]
Convulsions, not elsewhere classified				
First encounter: telehealth	1022 (24.8)	0.69 (0.59-0.81)	0.79 (0.53-1.18)	1.10 (0.77-1.57)
First encounter: in person	3099 (75.2)	1 [Reference]	1 [Reference]	1 [Reference]
Diseases of pulp and periapical tissues				
First encounter: telehealth	2480 (19.0)	1.01 (0.92-1.10)	0.98 (0.77-1.24)	0.92 (0.67-1.27)
First encounter: in person	10 570 (81.0)	1 [Reference]	1 [Reference]	1 [Reference]
Other diseases of lip and oral mucosa				
First encounter: telehealth	2271 (13.5)	0.96 (0.87-1.06)	1.01 (0.75-1.36)	1.01 (0.71-1.44)
First encounter: in person	14 573 (86.5)	1 [Reference]	1 [Reference]	1 [Reference]
Pneumonia, unspecified organism				
First encounter: telehealth	1402 (15.9)	1.68 (1.37-2.06)	1.07 (0.84-1.38)	0.76 (0.59-0.99)
First encounter: in person	7420 (84.1)	1 [Reference]	1 [Reference]	1 [Reference]
Stomatitis and related lesions				
First encounter: telehealth	2187 (15.1)	1.06 (0.96-1.17)	1.29 (1.00-1.65)	0.96 (0.69-1.35)
First encounter: in person	12 284 (84.9)	1 [Reference]	1 [Reference]	1 [Reference]
**Chronic ambulatory care sensitive conditions**				
Asthma				
First encounter: telehealth	23 878 (24.7)	0.98 (0.95-1.01)	0.98 (0.88-1.08)	0.92 (0.82-1.03)
First encounter: in person	72 918 (75.3)	1 [Reference]	1 [Reference]	1 [Reference]
Epilepsy and recurrent seizures				
First encounter: telehealth	3013 (28.3)	0.83 (0.76-0.91)	0.92 (0.69-1.22)	0.91 (0.68-1.20)
First encounter: in person	7645 (71.7)	1 [Reference]	1 [Reference]	1 [Reference]
Essential (primary) hypertension				
First encounter: telehealth	42 450 (16.5)	0.86 (0.84-0.88)	0.96 (0.88-1.05)	0.96 (0.89-1.03)
First encounter: in person	215 227 (83.5)	1 [Reference]	1 [Reference]	1 [Reference]
Heart failure				
First encounter: telehealth	556 (12.5)	0.63 (0.50-0.80)	0.86 (0.54-1.36)	1.05 (0.77-1.43)
First encounter: in person	3908 (87.5)	1 [Reference]	1 [Reference]	1 [Reference]
Hypertensive heart disease				
First encounter: telehealth	1259 (14.3)	0.76 (0.66-0.88)	0.89 (0.62-1.27)	1.11 (0.85-1.46)
First encounter: in person	7551 (85.7)	1 [Reference]	1 [Reference]	1 [Reference]
Iron deficiency anemia				
First encounter: telehealth	2812 (23.5)	1.24 (1.10-1.40)	0.99 (0.79-1.25)	0.89 (0.73-1.09)
First encounter: in person	9174 (76.5)	1 [Reference]	1 [Reference]	1 [Reference]
Other and unspecified noninfective gastroenteritis and colitis				
First encounter: telehealth	7586 (25.7)	1.28 (1.20-1.36)	1.09 (0.96-1.24)	0.93 (0.79-1.09)
First encounter: in person	21 929 (74.3)	1 [Reference]	1 [Reference]	1 [Reference]
Other chronic obstructive pulmonary disease				
First encounter: telehealth	1961 (17.6)	0.93 (0.83-1.04)	1.06 (0.82-1.37)	0.96 (0.76-1.20)
First encounter: in person	9165 (82.4)	1 [Reference]	1 [Reference]	1 [Reference]
Other disorders of teeth and supporting structures				
First encounter: telehealth	1479 (19.7)	1.01 (0.90-1.13)	0.83 (0.61-1.13)	1.15 (0.80-1.65)
First encounter: in person	6044 (80.3)	1 [Reference]	1 [Reference]	1 [Reference]
Type 1 diabetes mellitus				
First encounter: telehealth	689 (13.6)	0.73 (0.61-0.87)	1.12 (0.72-1.75)	0.74 (0.46-1.18)
First encounter: in person	4365 (86.4)	1 [Reference]	1 [Reference]	1 [Reference]
Type 2 diabetes mellitus				
First encounter: telehealth	8798 (14.3)	1.11 (1.05-1.17)	0.74 (0.61-0.90)	0.90 (0.78-1.03)
First encounter: in person	52 817 (85.7)	1 [Reference]	1 [Reference]	1 [Reference]

Abbreviations: ED, emergency department; OR, odds ratio.

^a^ The logistic regression models (2 separate models for acute and chronic ambulatory care sensitive conditions) included an interaction term between the type of initial encounter and the specific ambulatory care sensitive condition to address how telehealth effectiveness may vary by each clinical condition type. The models were adjusted for age, sex, Elixhauser Comorbidity Index, urban or rural status of residence, COVID-19 hotspot of residence, Area Deprivation Index, internet connectivity, severity index (for chronic conditions only), and the type of acute and chronic ambulatory care sensitive conditions. Refer to eTables 5 and 10 in the Supplement for more information on the approach to selecting acute and chronic ambulatory care sensitive conditions for the assessment of use patterns and the summary of acute and chronic ambulatory care sensitive conditions and subsequent utilization patterns during 2020.

When initial telehealth encounters were compared with initial in-person encounters within comparable 3-digit *ICD-10* disease categories, patients tended to have higher diagnostic severity levels. For example, 10.1% of telehealth encounters had a severity level of 2.0% vs 7.4% of in-person encounters (eTable 12 in the Supplement). This finding suggests the possibility that telehealth may be used preferentially for sicker patients, perhaps as a quicker way to treat patients with greater need.

## Discussion

A previous study^2^ examined the association of COVID-19 and changing coverage policies with the uptake of telehealth for a commercially insured population during the initial phase of the pandemic (March to June 2020). In this study, we extended the time frame to the next phase of the pandemic (July to December 2020). During this period of more established telehealth use, we undertook an analysis within our insured cohort that allowed us to assess follow-up encounters among comparable episodes of care delivered via telehealth vs in-person encounters.

### Major Study Findings

Our findings describe a period of continuity during the second half of 2020, after the marked changes that occurred during the first half of the year. From March to June 2020, there was initially a precipitous decrease in in-person ambulatory encounter rates accompanied by a marked increase in telehealth encounters.^2,28^ Clinicians and patients adapted quickly, and by the end of June, the persistent decrease in in-person office encounters was fully offset by a corresponding increase in telehealth encounters.^2^ This picture was similar to those identified in other reports^3,4,5,6,13,14,28,29,30,31^ on telehealth expansion in the early months of the COVID-19 pandemic.

In this study, we followed up a well-defined, continuously insured cohort of patients to offer an assessment of factors associated with changing patterns of telehealth use beyond the initial months of telehealth implementation.^12^ In contrast with the early months of the pandemic, patterns of use for July to December 2020 stabilized, with ambulatory encounters per health plan member being approximately the same for the summer of 2020 vs 2019. The mix of telehealth modalities (video, telephone, and other) also was relatively stable in March to December 2020.^2^ Although ambulatory encounters per member (in-person encounters plus telehealth equivalents) and the percentage of encounters via telehealth were relatively stable during the summer and fall of 2020, some subpopulation of members had lower encounter rates in 2020 compared with 2019. In 2020, the numbers of encounters for members aged 0 to 17 years were lower than comparable rates for 2019.^32^ Decreased well-child visits associated with lower 2020 birth rates may explain part of this difference.^33^ The telehealth rate for this age group also was lower than for the others, in part explaining the lower overall encounters. This result suggests that telehealth may not be viewed as equivalent to in-person encounters for younger patients or possibly for the type of problems that drive many pediatric encounters (eg, otitis media). These findings were similar to those detailed by Schweiberger et al,^32^ who identified a decreased number of pediatric care encounters because of fewer problem-focused encounters, with notably fewer infection-related encounters 7 months after the beginning of the pandemic. Ambulatory encounter rates were also decreased in the summer and fall of 2020 for older members (≥50 years of age) but to a lesser extent than for children and adolescents.

Encounter rates by the member and encounter characteristics illustrated that treatment patterns were modestly different in 2020 compared with 2019, despite the availability of telehealth as an alternative. Conversely, increased telehealth use in 2020 (summer and fall) compared with 2019 appeared to have prevented what would otherwise have been a precipitous decrease in ambulatory care triggered by COVID-19.

### Association With Outcomes of Care

We provide a comparison of key outcomes of care 14 days after initial telehealth vs in-person encounters for a set of acute and chronic ACS conditions. Although other studies^15,16^ have assessed such an association among a small sample of patients, we expanded this work and assessed the outcomes for acute and chronic ACS conditions separately and beyond in-person hospitalization. Our results showed that the use of telehealth services for the management of chronic ACS conditions appeared to be comparable to in-person encounters concerning the need for follow-up. However, patients with an initial telehealth encounter for 1 of the acute ACS conditions appeared to require additional follow-up compared with patients with an initial in-person ambulatory encounter.

From our analysis of episodes of care for specific presenting conditions, we identified that follow-up encounters after an index telehealth encounter were substantially more common for acute respiratory infections. Such increased follow-up was not more likely for other types of acute conditions (eg, acute pyelonephritis), suggesting that this difference could reflect concerns associated with the ongoing pandemic. Given that symptoms of these respiratory infections may be similar to those of COVID-19, one explanation of the higher number of follow-up encounters after an initial telehealth encounter of these types could reflect follow-up linked to suspected COVID-19 (eg, testing or ensuring adequate patient recovery). In contrast, follow-up care was generally less frequent after an initial telehealth encounter for a chronic condition than for an in-person encounter for the same condition.

### Limitations

This study has some limitations. Our results comparing the association of telehealth with follow-up care should be interpreted with caution. Although we applied multivariate regression modeling to account for a range of factors that could bias the results, some uncontrolled confounding bias might remain. One example would be the bias in the choice of telehealth and in-person encounter. For instance, a clinician may choose to provide a telehealth encounter to perform an initial assessment of a patient with mobility limitations before recommending an ED encounter or hospitalization. The finding of patients with complex clinical conditions (higher diagnostic severity levels) having more telehealth than in-person encounters support this concern. In addition, the end points used to compare telehealth and in-person encounters were limited in scope and temporality. Other clinically important end points for comparison would include the use of relevant laboratory tests and medication use and adjustments. Another alternative approach would be assessing additional models of care that integrate telehealth (eg, a telehealth and in-person hybrid model) and their effect on outcomes of care. Moreover, the time window for monitoring outcomes could be extended beyond 14 days, and a broader range of conditions could be studied. The nationally representative database we used did not include Medicare, Medicaid, or uninsured patients. Thus, experiences among these special needs patients could be different from those we documented in this study.

## Conclusions

In this cohort study of 40.7 million US commercially insured adults telehealth accounted for a large share of ambulatory encounters at the peak of the COVID-19 pandemic and remained prevalent after infection rates subsided. We identified many patient, practitioner, and community factors associated with the higher telehealth use, and trends were similar to those observed during the early months of the pandemic. Moreover, we compared the clinically relevant outcomes for telehealth vs in-person encounters in a nationally representative population, which extends our knowledge in terms of assessing the association of telehealth with outcomes of care. This study found that the use of telehealth services for the management of chronic ACS conditions was comparable, or even more efficient, than in-person care when follow-up encounters were assessed. On the other hand, patients with an initial telehealth encounter for acute ACS conditions appeared to require additional follow-up. This trend was observed especially for acute respiratory-related conditions, which potentially could be confounded by concerns over COVID-19 rather than the less complicated acute non–COVID-19 diagnosis. These findings suggest a direction for future work and are relevant to policy makers, payers, and practitioners as they manage the use of telehealth during the pandemic and afterward.

## Notes

Supplement. **eTable 1.** A Comprehensive List of Telehealth-Eligible Services and Associated Codes **eTable 2.** CMS Designated Codes for Place of Service **eTable 3.** Approach to Designating Telehealth Services (From Among Telehealth-Eligible Services) and Type of Modality **eTable 4.** Study Designated Specialty Using CMS Provider Specialty Codes **eTable 5.** Approach to Selecting Acute and Chronic Ambulatory Care Sensitive Conditions for Assessment of Utilization Patterns **eTable 6.** Severity Levels Assigned to ACS Chronic Condition *ICD-10* Codes within 3-Digit Code Categories **eTable 7.** Ambulatory Encounters and Percentage Telehealth During July-December 2019 and 2020: Unadjusted Results Comparing Full Population With Those Included in the Regression Analysis **eTable 8.** In-Person and Telehealth Ambulatory Encounters for Continuously Enrolled Insured Persons During Post Implementation Phase (July-December); Comparing Pre COVID-19 (2019) and Post COVID-19 (2020) Periods **eTable 9.** Ambulatory Encounters and Percentage Telehealth During 2019 and 2020: Unadjusted Results Broken Down by Characteristics of Persons and Their Residence Location **eTable 10.** Ambulatory Encounters and Percentage Telehealth During 2019 and 2020: Unadjusted Encounter Level Results Broken Down by Characteristics of the Encounter **eTable 11.** Summary of Acute and Chronic Ambulatory Care Sensitive Conditions and Subsequent Utilization Patterns During 2020 **eTable 12.** Counts and Percentage of Initial Ambulatory Encounters for Chronic Ambulatory Care Sensitive Conditions by Severity Level and Encounter Type **eFigure 1.** Sample Selection and Subgroup Identification Diagram **eFigure 2.** The ratio of 2020 to 2019 Ambulatory (AMB) Clinical Encounters by Week, March through June **eFigure 3.** Telehealth-Eligible Ambulatory (AMB) Encounters per 1000 Enrollees by Week, March-June 2019 and 2020
