# Supplementary material for: Outcomes of In-Person and Telehealth Ambulatory Encounters During COVID-19 Within a Large Commercially Insured Cohort
Source: JAMA Netw Open. 2022 Apr 26;5(4):e228954. doi: 10.1001/jamanetworkopen.2022.8954 (PMC9044109; doi:10.1001/jamanetworkopen.2022.8954)
Supplement: Supplement. — eTable 1. A Comprehensive List of Telehealth-Eligible Services and Associated Codes eTable 2. CMS Designated Codes for Place of Service eTable 3. Approach to Designating Telehealth Services (From Among Telehealth-Eligible Services) and Type of Modality eTable 4. Study Designated Specialty Using CMS Provider Specialty Codes eTable 5. Approach to Selecting Acute and Chronic Ambulatory Care Sensitive Conditions for Assessment of Utilization Patterns eTable 6. Severity Levels Assigned to ACS Chronic Condition ICD-10 Codes within 3-Digit Code Categories eTable 7. Ambulatory Encounters and Percentage Telehealth During July-December 2019 and 2020: Unadjusted Results Comparing Full Population With Those Included in the Regression Analysis eTable 8. In-Person and Telehealth Ambulatory Encounters for Continuously Enrolled Insured Persons During Post Implementation Phase (July-December); Comparing Pre COVID-19 (2019) and Post COVID-19 (2020) Periods eTable 9. Ambulatory Encounters and Percentage Telehealth During 2019 and 2020: Unadjusted Results Broken Down by Characteristics of Persons and Their Residence Location eTable 10. Ambulatory Encounters and Percentage Telehealth During 2019 and 2020: Unadjusted Encounter Level Results Broken Down by Characteristics of the Encounter eTable 11. Summary of Acute and Chronic Ambulatory Care Sensitive Conditions and Subsequent Utilization Patterns During 2020 eTable 12. Counts and Percentage of Initial Ambulatory Encounters for Chronic Ambulatory Care Sensitive Conditions by Severity Level and Encounter Type eFigure 1. Sample Selection and Subgroup Identification Diagram eFigure 2. The ratio of 2020 to 2019 Ambulatory (AMB) Clinical Encounters by Week, March through June eFigure 3. Telehealth-Eligible Ambulatory (AMB) Encounters per 1000 Enrollees by Week, March-June 2019 and 2020 [file jamanetwopen-e228954-s001.pdf]

# Supplementary Online Content

Hatef E, Lans D, Bandeian S, Lasser EC, Goldsack J, Weiner JP. Outcomes of in-person telehealth ambulatory encounters during COVID-19 within a large commercially insured cohort. *JAMA Netw Open*. 2022;5(4):e228954. doi:10.1001/jamanetworkopen.2022.8954

**eTable 1.** A Comprehensive List of Telehealth-Eligible Services and Associated Codes

**eTable 2.** CMS Designated Codes for Place of Service

**eTable 3.** Approach to Designating Telehealth Services (From Among Telehealth-Eligible Services) and Type of Modality

**eTable 4.** Study Designated Specialty Using CMS Provider Specialty Codes

**eTable 5.** Approach to Selecting Acute and Chronic Ambulatory Care Sensitive Conditions for Assessment of Utilization Patterns

**eTable 6.** Severity Levels Assigned to ACS Chronic Condition *ICD-10* Codes within 3-Digit Code Categories

**eTable 7.** Ambulatory Encounters and Percentage Telehealth During July-December 2019 and 2020: Unadjusted Results Comparing Full Population With Those Included in the Regression Analysis

**eTable 8.** In-Person and Telehealth Ambulatory Encounters for Continuously Enrolled Insured Persons During Post Implementation Phase (July-December); Comparing Pre-COVID-19 (2019) and Post-COVID-19 (2020) Periods

**eTable 9.** Ambulatory Encounters and Percentage Telehealth During 2019 and 2020: Unadjusted Results Broken Down by Characteristics of Persons and Their Residence Location

**eTable 10.** Ambulatory Encounters and Percentage Telehealth During 2019 and 2020: Unadjusted Encounter Level Results Broken Down by Characteristics of the Encounter

**eTable 11.** Summary of Acute and Chronic Ambulatory Care Sensitive Conditions and Subsequent Utilization Patterns During 2020

**eTable 12.** Counts and Percentage of Initial Ambulatory Encounters for Chronic Ambulatory Care Sensitive Conditions by Severity Level and Encounter Type

**eFigure 1.** Sample Selection and Subgroup Identification Diagram

**eFigure 2.** The ratio of 2020 to 2019 Ambulatory (AMB) Clinical Encounters by Week, Marchthrough June

**eFigure 3.** Telehealth-Eligible Ambulatory (AMB) Encounters per 1000 Enrollees by Week, March-June 2019 and 2020

**This supplementary material has been provided by the authors to give readers additional information about their work.**

© 2022 Hatef E et al. *JAMA Network Open.*

## eTable 1: A Comprehensive List of Telehealth-Eligible Services and Associated Codes[a]

| Type of Service | CPT and HCPCS Codes associated with Type of Service |
|---|---|
| **E & M – office** | 99201, 99202, 99203, 99204, 99205, 99211, 99212, 99213, 99214, 99215, 99354, 99355, 99421 [c], 99422 [c], 99423 [c], 99441 [c], 99442 [c], 99443 [c], G2012, G2061 [c], G2062 [c], G2063 [c] |
| **E & M – emergency department [b]** | 99281, 99282, 99283, 99284, 99285 |
| **E & M – hospital [b]** | 99217, 99218, 99219, 99220, 99221, 99222, 99223, 99224, 99225, 99226, 99231, 99232, 99233, 99234, 99235, 99236, 99238, 99239, 99291, 99292, 99356, 99357, 99468, 99469, 99471, 99472, 99475, 99476, 99477, 99478, 99479, 99480, G0508 [c], G0509 [c] |
| **Behavioral health** | 0362T, 0373T, 90785, 90791, 90792, 90832, 90833, 90834, 90836, 90837, 90838, 90839, 90840, 90845, 90846, 90847, 90853, 90875, 97151, 97152, 97153, 97154, 97155, 97156, 97157, 97158, G0396, G0397, G0410, G0443,G2086, G2087, G2088 |
| **Rehabilitation** | 97110, 97112, 97116, 97150, 97161, 97162, 97163, 97164, 97165, 97166, 97167, 97168, 97530, 97535, 97542, 97750, 97755, 97760, 97761, S9152 |
| **Other** | 77427, 90951, 90952, 90953, 90954, 90955 90956, 90957, 90958, 90959, 90960, 90961, 90962, 90963, 90964, 90965, 90966, 90967, 90968, 90969, 90970, 92002, 92004, 92012, 92014, 92507, 92508, 92521, 92522, 92523, 92524, 92601, 92602, 92603, 92604, 94002, 94003, 94004, 94005, 94664, 96110, 96112, 96113, 96116, 96121, 96127, 96130, 96131, 96132, 96133, 96136, 96137, 96138, 96139, 96156, 96158, 96159, 96160, 96161, 96164, 96165, 96167, 96168, 96170, 96171, 97802, 97803, 97804, 98966 [c], 98967 [c], 98968 [c], 98970 [c], 98971 [c], 98972 [c], 99091 [c], 99304, 99305, 99306, 99307, 99308, 99309, 99310, 99315, 99316, 99324, 99325, 99326, 99327, 99328, 99334, 99335, 99336, 99337, 99341, 99342, 99343, 99344, 99345, 99347, 99348, 99349, 99350, 99406, 99407, 99446 [c], 99447 [c], 99448 [c], 99449 [c], 99451 [c], 99452 [c], 99453 [c], 99454 [c], 99457 [c], 99458 [c], 99473, 99474 [c], 99483, 99495, 99496, 99497, 99498, G0108, G0109, G0270, G0296, G0406 [c], G0407 [c], G0408 [c], G0420, G0421, G0425 [c], G0426 [c], G0427 [c],G0436, G0437,G0438, G0439, G0442, G0444, G0445, G0446, G0447, G0459 [c], G0506, G0513, G0514, G2010 [c], G9685, Q3014 [c], T1014 [c] |

[A] A list of CPT® and HCPCS codes for telehealth-eligible services by type of service (source: CMS List of telehealth services. https://www.cms.gov/Medicare/Medicare-General-Information/Telehealth/Telehealth-Codes)

[b] Type of service was excluded from our study.

[c] Code does not require a modifier or place of service code if provided via telehealth.

CPT: Current Procedural Terminology, E & M: means evaluation and management, HCPCS: HealthcareCommon Procedure Coding System

© 2022 Hatef E et al. *JAMA Network Open.*

## eTable 2. CMS Designated Codes for Place of Service

| Place of Service Category | CMS Place of Service Code | CMS Place of Service Name |
|---|---|---|
| **Inpatient** | 13 | Assisted Living Facility |
| | 14 | Group Home |
| | 21 | Inpatient Hospital |
| | 31 | Skilled Nursing Facility |
| | 32 | Nursing Facility |
| | 33 | Custodial Care Facility |
| | 51 | Inpatient Psychiatric Facility |
| | 54 | Intermediate Care Facility |
| | 55 | Residential Substance Abuse Treatment Facility |
| | 56 | Psychiatric Residential Treatment Center |
| | 61 | Comprehensive Inpatient Rehabilitation Facility |
| **Emergency Department** | 23 | Emergency Room – Hospital |
| **Outpatient Facility** | 05 | Indian Health Service Free-standing Facility |
| | 06 | Indian Health Service Provider-based Facility |
| | 07 | Tribal 638 Free-standing Facility |
| | 08 | Tribal 638 Provider-based Facility |
| | 19 | Off Campus-Outpatient Hospital |
| | 22 | On Campus-Outpatient Hospital |
| | 24 | Ambulatory Surgical Center |
| | 25 | Birthing Center |
| | 26 | Military Treatment Facility |
| | 52 | Psychiatric Facility-Partial Hospitalization |
| | 57 | Non-residential Substance Abuse Treatment Facility |
| | 58 | Non-residential Opioid Treatment Facility |
| | 62 | Comprehensive Outpatient Rehabilitation Facility |
| | 65 | End-Stage Renal Disease Treatment Facility |
| **Office / Clinic** | 11 | Office |
| | 17 | Walk-in Retail Health Clinic |
| | 18 | Place of Employment-Worksite |
| | 20 | Urgent Care Facility |
| | 49 | Independent Clinic |
| | 50 | Federally Qualified Health Center |
| | 53 | Community Mental Health Center |
| | 60 | Mass Immunization Center |
| | 71 | Public Health Clinic |
| | 72 | Rural Health Clinic |
| **Telehealth** | 02 | Telehealth |
| **Other Ambulatory** | 01 | Pharmacy |
| | 03 | School |
| | 04 | Homeless Shelter |
| | 09 | Prison/ Correctional Facility |
| | 12 | Home |
| | 15 | Mobile Unit |
| | 16 | Temporary Lodging |
| | 34 | Hospice |

© 2022 Hatef E et al. *JAMA Network Open.*

| | | |
|---|---|---|
| | 41 | Ambulance - Land |
| | 42 | Ambulance – Air or Water |
| | 81 | Independent Laboratory |
| | 99 | Other Place of Service |
| | UN | Unknown Place of Service |
| CMS: Centers for Medicare and Medicaid Services | | |

**eTable 3. Approach to Designating Telehealth Services (from Among Telehealth Eligible Services) and Type of Modality**
*(See text for further explanation of methodology)*

## A: Service Modifier and Place of Service Codes Used to Designate Telehealth Service and Modality

| Code Type | Code | Definition | Modality Classification |
|---|---|---|---|
| **Modifier** | GT | "Via interactive audio and video telecommunications systems." | Video-Supported telehealth |
| | 95 | "Synchronous telemedicine service rendered via a real-time interactive audioand video telecommunications system." | Video Supported-Telehealth |
| **Place of Service** | 02 | The location where health services and health-related services are provided or received, through a telecommunication system. | Telehealth - not specified |
| Telehealth was defined as one of the telehealth-eligible codes with a GT or 95 modifiers or place of service code '02. | | | |

## B: Assignment of Telehealth Eligible Service Codes to Specific Type of Virtual Encounter Modality

| |
|---|
| **Remote monitoring** |
| **99457**, **99458** |
| **Video supported telehealth** |
| **G0425**, **G0426**, **G0427, G0508**, **G0509** |
| **Telephone** |
| **98966**, **98967**, **98968**, **99441**, **99442**, **99443** |
| An encounter with any of the listed codes was considered as telehealth, regardless of modifier status and place of service. |

## eTable 4. Study Designated Specialty Using CMS Provider Specialty Codes

| Study Designated Specialty | CMS Provider Specialty Code | Specialty Description |
|---|---|---|
| **Primary Care** | 1 | GENERAL PRACTICE |
| | 8 | FAMILY PRACTICE |
| | 11 | INTERNAL MEDICINE |
| | 16 | OBSTETRICS GYNECOLOGY |
| | 37 | PEDIATRIC MEDICINE |
| | 38 | GERIATRIC MEDICINE |
| **Medical Specialist** | 3 | ALLERGY / IMMUNOLOGY |
| | 6 | CARDIOLOGY |
| | 7 | DERMATOLOGY |
| | 10 | GASTROENTEROLOGY |
| | 13 | NEUROLOGY |
| | 21 | CARDIAC ELECTROPHYSIOLOGY |
| | 29 | PULMONARY DISEASE |
| | 39 | NEPHROLOGY |
| | 44 | INFECTIOUS DISEASES |
| | 46 | ENDOCRINOLOGY |
| | 66 | RHEUMATOLOGY |
| | 81 | CRITICAL CARE (INTENSIVISTS) |
| | 82 | HEMATOLOGY |
| | 83 | HEMATOLOGY / ONCOLOGY |
| | 84 | PREVENTIVE MEDICINE |
| | 90 | MEDICAL ONCOLOGY |
| | C0 | SLEEP MEDICINE |
| **Surgical Specialist** | 2 | GENERAL SURGERY |
| | 4 | OTOLARYNGOLOGY |
| | 14 | NEUROSURGERY |
| | 18 | OPTHALMOLOGY |
| | 19 | ORAL SURGERY (DENTISTS ONLY) |
| | 20 | ORTHOPEDIC SURGERY |
| | 23 | SPORTS MEDICINE |
| | 24 | PLASTIC AND RECONSTRUCTIVE SURGERY |
| | 28 | COLORECTAL SURGERY (FORMERLY PROCTOLOGY) |
| | 33 | THORACIC SURGERY |
| | 34 | UROLOGY |
| | 40 | HAND SURGERY |
| | 76 | PERIPHERAL VASCULAR DISEASE |
| | 77 | VASCULAR SURGERY |
| | 78 | CARDIAC SURGERY |
| | 85 | MAXILLOFACIAL SURGERY |
| | 91 | SURGICAL ONCOLOGY |
| | 98 | GYNECOLOGICAL / ONCOLOGY |
| **Behavioral Health** | 26 | PSYCHIATRY |
| | 27 | GERIATRIC PSYCHIATRY |
| | 62 | INDEPENDENT BILLING PSYCHOLOGIST |

© 2022 Hatef E et al. *JAMA Network Open.*

| | | |
|---|---|---|
| | 68 | CLINICAL PSYCHOLOGIST |
| | 79 | ADDICTION MEDICINE |
| | 86 | NEUROPSYCHIATRY |
| **Rehabilitation** | 12 | OSTEOPATHIC MANIPULATIVE THERAPY |
| | 15 | SPEECH LANGUAGE PATH. IN PRIV. PRACTICE |
| | 25 | PHYSICAL MEDICINE AND REHABILITATION |
| | 35 | CHIROPRACTIC |
| | 65 | INDEPENDENTLY PRACTICING PHYSICAL THERAPIST |
| | 67 | INDEPEND. PRACTICING OCCUPATIONAL THERAPIST |
| **Physician Assistant / Nurse Practitioner** | 50 | NURSE PRACTITIONER |
| | 89 | CERTIFIED CLINICAL NURSE SPECIALIST |
| | 97 | PHYSICIAN ASSISTANT |
| **Other Physician / Practitioner** | 5 | ANESTHESIOLOGY |
| | 9 | INTERVENTIONAL PAIN MANAGEMENT |
| | 22 | PATHOLOGY |
| | 30 | DIAGNOSTIC RADIOLOGY |
| | 32 | ANESTHESIOLOGIST ASSISTANTS |
| | 36 | NUCLEAR MEDICINE |
| | 41 | OPTOMETRY |
| | 42 | CERTIFIED NURSE MIDWIFE |
| | 43 | CERTIFIED RN ANESTHETIST, ANESTHESIA ASSISTANT |
| | 48 | PODIATRY |
| | 55 | INDIVIDUAL CERTIFIED ORTHOTIST |
| | 56 | INDIVIDUAL CERTIFIED PROSTHETIST |
| | 57 | INDIVIDUAL CERTIFIED PROSTHETIST-ORTHOTIST |
| | 64 | INDEPENDENTLY BILLING AUDIOLOGIST |
| | 70 | MULTI SPECIALTY CLINIC OR GROUP PRACTICE |
| | 71 | REGISTERED DIETICIAN/NUTRITION PROFESSIONAL |
| | 72 | PAIN MANAGEMENT |
| | 80 | LICENSED CLINICAL SOCIAL WORKER |
| | 92 | RADIATION ONCOLOGY |
| | 93 | EMERGENCY MEDICINE |
| | 94 | INTERVENTIONAL RADIOLOGY |
| | 96 | OPTICIAN |
| | 99 | UNKNOWN PHYSICIAN SPECIALTY |
| | B2 | PEDORTHIC PERSONNEL |
| | B5 | OCULARIST |
| **Other** | 51 | MEDICAL SUPPLY COMP.WITH CERTIFIED ORTHOTIST |
| | 52 | MEDICAL SUPPLY COMP. W/CERTIFIED PROSTHETIST |
| | 53 | MED SUPPLY CO. W/CERT. PROSTHETIST ORTHOTIST |
| | 54 | MEDICAL SUPPLY COMPANY NOT 51, 52, OR 53 |
| | 58 | MEDICAL SUPPLY COMP. W/REGIST. PHARMACIST |
| | 59 | AMBULANCE SERVICE SUPPLIER |

© 2022 Hatef E et al. *JAMA Network Open.*

| | | |
|---|---|---|
| | 60 | PUBLIC HEALTH OR WELFARE AGENCIES |
| | 61 | VOLUNTARY HEALTH OR CHARITABLE AGENCIES |
| | 63 | PORTABLE X-RAY SUPPLIER |
| | 69 | INDEPENDENTLY BILLING CLINICAL LABORATORY |
| | 73 | MASS IMMUNIZATION ROSTER BILLER |
| | 75 | SLIDE PREPARATION FACILITIES |
| | 87 | ALL OTHER SUPPLIERS |
| | 88 | UNKNOWN SUPPLIER |
| | A4 | HOME HEALTH AGENCY |
| | A5 | PHARMACY |
| | A6 | MEDICAL SUPPLY COMP. W/RESPIRATORY THERAPIST |
| | A7 | DEPARTMENT STORE |
| | A8 | GROCERY STORE |
| | B3 | MEDICAL SUPPLY CO W/ PEDORTHIC PERSONNEL |
| | B4 | REHABILITATION AGENCY |
| | C1 | CENTRALIZED FLU |
| | IN | INVALID |
| **Facility** | 17 | HOSPICE AND PALLIATIVE CARE |
| | 31 | INTENSIVE CARDIAC REHABILITATION (ICR) |
| | 45 | MAMMOGRAPHY SCREENING CENTER |
| | 47 | INDEPENDENT DIAGNOSTIC TESTING FACILITY |
| | 49 | AMBULATORY SURGICAL CENTER |
| | 74 | RADIATION THERAPY CENTER |
| | A0 | HOSPITAL |
| | A1 | SKILLED NURSING FACILITY |
| | A2 | INTERMEDIATE CARE NURSING FACILITY |
| | A3 | NURSING FACILITY, OTHER |

© 2022 Hatef E et al. *JAMA Network Open.*

**eTable 5. Approach to Selecting Acute and Chronic Ambulatory Care Sensitive Conditions for Assessment of Utilization Patterns**. *(See text for further explanation of methodology)*

| Clinical Condition | Description | No. Members | No. Ambulatory Encounters | % of Total Ambulatory Encounters via Telehealth |
|---|---|---|---|---|
| I10 | Essential (Primary) Hypertension | 1,996,347 | 2,729,223 | 321,812 (11.79%) |
| E11 | Type 2 Diabetes Mellitus | 1,152,835 | 1,906,344 | 243,223 (12.76%) |
| J02 | Acute Pharyngitis | 638,249 | 704,672 | 91,253 (12.95%) |
| J06 | Acute Upper Resp Infections of Multiple and Unspecified Sits | 522,687 | 566,762 | 100,677 (17.76%) |
| J45 | Asthma | 395,684 | 512,657 | 95,551 (18.64%) |
| E10 | Type 1 Diabetes Mellitus | 138,302 | 251,836 | 44,893 (17.83%) |
| D50 | Iron Deficiency Anemia | 89,378 | 127,502 | 18,578 (14.57%) |
| G40 | Epilepsy and Recurrent Seizures | 85,857 | 125,378 | 36,819 (29.37%) |
| J20 | Acute Bronchitis | 84,734 | 92,908 | 18,158 (19.54%) |
| K52 | Other and Unspecified Noninfective Gastroenteritis and Colitis | 69,451 | 81,153 | 17,951 (22.12%) |
| J44 | Other Chronic Obstructive Pulmonary Disease | 69,421 | 98,745 | 14,694 (14.88%) |
| J03 | Acute Tonsillitis | 53,588 | 59,352 | 6,323 (10.65%) |
| I50 | Heart Failure | 41,671 | 68,215 | 8,172 (11.98%) |
| I11 | Hypertensive Heart Disease | 41,517 | 55,274 | 6,627 (11.99%) |
| J18 | Pneumonia, Unspecified Organism | 34,355 | 45,025 | 5,809 (12.9%) |
| K13 | Other Diseases of Lip and Oral Mucosa | 28,598 | 32,629 | 3,466 (10.62%) |
| R56 | Convulsions, not Elsewhere Classified | 27,744 | 38,413 | 8,211 (21.38%) |
| K04 | Diseases of Pulp and Periapical Tissues | 18,435 | 19,573 | 3,657 (18.68%) |
| K08 | Other Disorders of Teeth and Supporting Structures | 10,340 | 10,839 | 2,112 (19.49%) |
| E16 | Other Disorders of Pancreatic Internal Secretion | 9,129 | 11,497 | 2,204 (19.17%) |
| N12 | Tubulo-interstitial Nephritis, Not Specified as Acute or Chronic | 6,418 | 7,822 | 834 (10.66%) |
| N10 | Acute Pyelonephritis | 6,411 | 7,800 | 842 (10.79%) |

We assessed the ambulatory encounters for each set of 3-digit ICD-10 codes for ACS conditions. From the cohort of members that had 1+ encounter during the study period, we counted the number of members that had an encounter for an avoidable ACS condition. As an initial screening process to eliminate rare conditions, we limited the list to conditions that were diagnosed in at least 5000 members. To ensure adequate use of telehealth among the conditions, we also required at least 10% of the members diagnosed with an ACS condition to have at least 1 telehealth claim associated with the condition. After screening for candidate conditions, additional inclusion criteria required members to have no pre-existing care related to the condition (no encounters for the condition 90 days before the initial encounter in the study period).

© 2022 Hatef E et al. *JAMA Network Open.*

**eTable 6. Severity Levels Assigned to ACS Chronic Condition *ICD-10* Codes within 3-Digit Code Categories**

| 3-digit ICD-10 | 3-digit ICD-10 description | ICD-10 | ICD-10 Description | Severity |
|---|---|---|---|---|
| D50 | Iron deficiency anemia | D500 | Iron deficiency anemia secondary to blood loss (chronic) | 1 |
| | | D501 | Sideropenic dysphagia | 1 |
| | | D508 | Other iron deficiency anemias | 1 |
| | | D509 | Iron deficiency anemia, unspecified | 1 |
| E10 | Type 1 diabetes mellitus | E1010 | Type 1 diabetes mellitus with ketoacidosis without coma | 3 |
| | | E1011 | Type 1 diabetes mellitus with ketoacidosis with coma | 3 |
| | | E10610 | Type 1 diabetes mellitus w diabetic neuropathic arthropathy | 2 |
| | | E10618 | Type 1 diabetes mellitus with other diabetic arthropathy | 2 |
| | | E10620 | Type 1 diabetes mellitus with diabetic dermatitis | 2 |
| | | E10621 | Type 1 diabetes mellitus with foot ulcer | 2 |
| | | E10622 | Type 1 diabetes mellitus with other skin ulcer | 2 |
| | | E10628 | Type 1 diabetes mellitus with other skin complications | 2 |
| | | E10630 | Type 1 diabetes mellitus with periodontal disease | 2 |
| | | E10638 | Type 1 diabetes mellitus with other oral complications | 2 |
| | | E10641 | Type 1 diabetes mellitus with hypoglycemia with coma | 3 |
| | | E10649 | Type 1 diabetes mellitus with hypoglycemia without coma | 2 |
| | | E1065 | Type 1 diabetes mellitus with hyperglycemia | 2 |
| | | E1069 | Type 1 diabetes mellitus with other specified complication | 2 |
| | | E108 | Type 1 diabetes mellitus with unspecified complications | 2 |
| | | E109 | Type 1 diabetes mellitus without complications | 1 |
| E11 | Type 2 diabetes mellitus | E1100 | Type 2 diab w hyprosm w/o nonket hyprgly-hypros coma (NKHHC) | 3 |
| | | E1101 | Type 2 diabetes mellitus with hyperosmolarity with coma | 3 |
| | | E11610 | Type 2 diabetes mellitus w diabetic neuropathic arthropathy | 2 |
| | | E11618 | Type 2 diabetes mellitus with other diabetic arthropathy | 2 |
| | | E11620 | Type 2 diabetes mellitus with diabetic dermatitis | 2 |
| | | E11621 | Type 2 diabetes mellitus with foot ulcer | 2 |
| | | E11622 | Type 2 diabetes mellitus with other skin ulcer | 2 |

© 2022 Hatef E et al. *JAMA Network Open.*

| 3-digit ICD-10 | 3-digit ICD-10 description | ICD-10 | ICD-10 Description | Severity |
|---|---|---|---|---|
| | | E11628 | Type 2 diabetes mellitus with other skin complications | 2 |
| | | E11630 | Type 2 diabetes mellitus with periodontal disease | 2 |
| | | E11638 | Type 2 diabetes mellitus with other oral complications | 2 |
| | | E11641 | Type 2 diabetes mellitus with hypoglycemia with coma | 3 |
| | | E11649 | Type 2 diabetes mellitus with hypoglycemia without coma | 2 |
| | | E1165 | Type 2 diabetes mellitus with hyperglycemia | 2 |
| | | E1169 | Type 2 diabetes mellitus with other specified complication | 2 |
| | | E118 | Type 2 diabetes mellitus with unspecified complications | 2 |
| | | E119 | Type 2 diabetes mellitus without complications | 1 |
| G40 | Epilepsy and recurrent seizures | G40001 | Local-rel idio epi w seiz of loc onst, not ntrct, w stat epi | 3 |
| | | G40009 | Local-rel idio epi w seiz of loc onst,not ntrct,w/o stat epi | 2 |
| | | G40011 | Local-rel idio epi w seiz of loc onset, ntrct, w stat epi | 3 |
| | | G40019 | Local-rel idio epi w seiz of loc onset, ntrct, w/o stat epi | 2 |
| | | G40101 | Local-rel symptc epi w simp part seiz, not ntrct, w stat epi | 3 |
| | | G40109 | Local-rel symptc epi w simp prt seiz,not ntrct, w/o stat epi | 2 |
| | | G40111 | Local-rel symptc epi w simple part seiz, ntrct, w stat epi | 3 |
| | | G40119 | Local-rel symptc epi w simple part seiz, ntrct, w/o stat epi | 2 |
| | | G40201 | Local-rel symptc epi w cmplx prt seiz, not ntrct, w stat epi | 3 |
| | | G40209 | Local-rel symptc epi w cmplx prt seiz,not ntrct,w/o stat epi | 2 |
| | | G40211 | Local-rel symptc epi w cmplx partial seiz, ntrct, w stat epi | 3 |
| | | G40219 | Local-rel symptc epi w cmplx part seiz, ntrct, w/o stat epi | 2 |
| | | G40301 | Gen idiopathic epilepsy, not intractable, w stat epi | 3 |
| | | G40309 | Gen idiopathic epilepsy, not intractable, w/o stat epi | 1 |
| | | G40311 | Generalized idiopathic epilepsy, intractable, w stat epi | 3 |
| | | G40319 | Generalized idiopathic epilepsy, intractable, w/o stat epi | 2 |
| | | G40401 | Oth generalized epilepsy, not intractable, w stat epi | 3 |
| | | G40409 | Oth generalized epilepsy, not intractable, w/o stat epi | 1 |
| | | G40411 | Oth generalized epilepsy, intractable, w status epilepticus | 3 |

© 2022 Hatef E et al. *JAMA Network Open.*

| 3-digit ICD-10 | 3-digit ICD-10 description | ICD-10 | ICD-10 Description | Severity |
|---|---|---|---|---|
| | | G40419 | Oth generalized epilepsy, intractable, w/o stat epi | 2 |
| | | G4042 | Cyclin-Dependent Kinase-Like 5 Deficiency Disorder | 1 |
| | | G40501 | Epileptic seiz rel to extrn causes, not ntrct, w stat epi | 3 |
| | | G40509 | Epileptic seiz rel to extrn causes, not ntrct, w/o stat epi | 2 |
| | | G40801 | Other epilepsy, not intractable, with status epilepticus | 3 |
| | | G40802 | Other epilepsy, not intractable, without status epilepticus | 2 |
| | | G40803 | Other epilepsy, intractable, with status epilepticus | 3 |
| | | G40804 | Other epilepsy, intractable, without status epilepticus | 2 |
| | | G40811 | Lennox-Gastaut syndrome, not intractable, w stat epi | 3 |
| | | G40812 | Lennox-Gastaut syndrome, not intractable, w/o stat epi | 2 |
| | | G40813 | Lennox-Gastaut syndrome, intractable, w status epilepticus | 3 |
| | | G40814 | Lennox-Gastaut syndrome, intractable, w/o status epilepticus | 2 |
| | | G40821 | Epileptic spasms, not intractable, with status epilepticus | 3 |
| | | G40822 | Epileptic spasms, not intractable, w/o status epilepticus | 2 |
| | | G40823 | Epileptic spasms, intractable, with status epilepticus | 3 |
| | | G40824 | Epileptic spasms, intractable, without status epilepticus | 2 |
| | | G40833 | Dravet syndrome, intractable, with status epilepticus | 3 |
| | | G40834 | Dravet syndrome, intractable, without status epilepticus | 2 |
| | | G4089 | Other seizures | 2 |
| | | G40901 | Epilepsy, unsp, not intractable, with status epilepticus | 3 |
| | | G40909 | Epilepsy, unsp, not intractable, without status epilepticus | 1 |
| | | G40911 | Epilepsy, unspecified, intractable, with status epilepticus | 3 |
| | | G40919 | Epilepsy, unsp, intractable, without status epilepticus | 2 |
| | | G40A01 | Absence epileptic syndrome, not intractable, w stat epi | 3 |
| | | G40A09 | Absence epileptic syndrome, not intractable, w/o stat epi | 1 |
| | | G40A11 | Absence epileptic syndrome, intractable, w stat epi | 3 |
| | | G40A19 | Absence epileptic syndrome, intractable, w/o stat epi | 2 |
| | | G40B01 | Juvenile myoclonic epilepsy, not intractable, w stat epi | 3 |
| | | G40B09 | Juvenile myoclonic epilepsy, not intractable, w/o stat epi | 2 |

© 2022 Hatef E et al. *JAMA Network Open.*

| 3-digit ICD-10 | 3-digit ICD-10 description | ICD-10 | ICD-10 Description | Severity |
|---|---|---|---|---|
| | | G40B11 | Juvenile myoclonic epilepsy, intractable, w stat epi | 3 |
| | | G40B19 | Juvenile myoclonic epilepsy, intractable, w/o stat epi | 2 |
| I10 | Essential (primary) hypertension | I10 | Essential (primary) hypertension | 1 |
| I11 | Hypertensive heart disease | I110 | Hypertensive heart disease with heart failure | 2 |
| | | I119 | Hypertensive heart disease without heart failure | 1 |
| I50 | Heart failure | I501 | Left ventricular failure, unspecified | 2 |
| | | I5020 | Unspecified systolic (congestive) heart failure | 2 |
| | | I5021 | Acute systolic (congestive) heart failure | 3 |
| | | I5022 | Chronic systolic (congestive) heart failure | 2 |
| | | I5023 | Acute on chronic systolic (congestive) heart failure | 3 |
| | | I5030 | Unspecified diastolic (congestive) heart failure | 2 |
| | | I5031 | Acute diastolic (congestive) heart failure | 3 |
| | | I5032 | Chronic diastolic (congestive) heart failure | 2 |
| | | I5033 | Acute on chronic diastolic (congestive) heart failure | 3 |
| | | I5040 | Unsp combined systolic and diastolic (congestive) hrt fail | 2 |
| | | I5041 | Acute combined systolic and diastolic (congestive) hrt fail | 3 |
| | | I5042 | Chronic combined systolic and diastolic hrt fail | 2 |
| | | I5043 | Acute on chronic combined systolic and diastolic hrt fail | 3 |
| | | I50810 | Right heart failure, unspecified | 1 |
| | | I50811 | Acute right heart failure | 2 |
| | | I50812 | Chronic right heart failure | 1 |
| | | I50813 | Acute on chronic right heart failure | 2 |
| | | I50814 | Right heart failure due to left heart failure | 1 |
| | | I5082 | Biventricular heart failure | 1 |
| | | I5083 | High output heart failure | 1 |
| | | I5084 | End stage heart failure | 2 |
| | | I5089 | Other heart failure | 1 |
| | | I509 | Heart failure, unspecified | 1 |
| J44 | Other chronic obstructive pulmonary disease | J440 | Chr obstructive pulmon disease with (acute) lower resp infct | 3 |
| | | J441 | Chronic obstructive pulmonary disease w (acute) exacerbation | 2 |

© 2022 Hatef E et al. *JAMA Network Open.*

| 3-digit ICD-10 | 3-digit ICD-10 description | ICD-10 | ICD-10 Description | Severity |
|---|---|---|---|---|
| | | J449 | Chronic obstructive pulmonary disease, unspecified | 1 |
| J45 | Asthma | J4520 | Mild intermittent asthma, uncomplicated | 1 |
| | | J4521 | Mild intermittent asthma with (acute) exacerbation | 2 |
| | | J4522 | Mild intermittent asthma with status asthmaticus | 3 |
| | | J4530 | Mild persistent asthma, uncomplicated | 1 |
| | | J4531 | Mild persistent asthma with (acute) exacerbation | 2 |
| | | J4532 | Mild persistent asthma with status asthmaticus | 3 |
| | | J4540 | Moderate persistent asthma, uncomplicated | 2 |
| | | J4541 | Moderate persistent asthma with (acute) exacerbation | 2 |
| | | J4542 | Moderate persistent asthma with status asthmaticus | 3 |
| | | J4550 | Severe persistent asthma, uncomplicated | 2 |
| | | J4551 | Severe persistent asthma with (acute) exacerbation | 3 |
| | | J4552 | Severe persistent asthma with status asthmaticus | 3 |
| | | J45901 | Unspecified asthma with (acute) exacerbation | 2 |
| | | J45902 | Unspecified asthma with status asthmaticus | 3 |
| | | J45909 | Unspecified asthma, uncomplicated | 1 |
| | | J45990 | Exercise induced bronchospasm | 1 |
| | | J45991 | Cough variant asthma | 1 |
| | | J45998 | Other asthma | 1 |
| K52 | Other and unsp noninfective gastroenteritis and colitis | K520 | Gastroenteritis and colitis due to radiation | 2 |
| | | K521 | Toxic gastroenteritis and colitis | 2 |
| | | K5221 | Food protein-induced enterocolitis syndrome | 2 |
| | | K5222 | Food protein-induced enteropathy | 2 |
| | | K5229 | Other allergic and dietetic gastroenteritis and colitis | 2 |
| | | K523 | Indeterminate colitis | 2 |
| | | K5281 | Eosinophilic gastritis or gastroenteritis | 2 |
| | | K5282 | Eosinophilic colitis | 2 |
| | | K52831 | Collagenous colitis | 2 |
| | | K52832 | Lymphocytic colitis | 2 |
| | | K52838 | Other microscopic colitis | 2 |
| | | K52839 | Microscopic colitis, unspecified | 2 |
| | | K5289 | Other specified noninfective gastroenteritis and colitis | 2 |
| | | K529 | Noninfective gastroenteritis and colitis, unspecified | 1 |

© 2022 Hatef E et al. *JAMA Network Open.*

**eTable 7. Ambulatory Encounters and Percentage Telehealth During July-December 2019 and 2020: Unadjusted Results Comparing Full Population with Those Included in the Regression Analysis**

| Patient Level Characteristics | Full Population Patient No. (%) | Regression Analysis Cohort Patient No. (%) |
|---|---|---|
| **Total No. Members** | **40,739,915** | **1,086,720** |
| **Age in 2019** | | |
| 0 to 17 | 9,102,907 (22.34%) | 211,124 (19.43%) |
| 18 to 34 | 9,755,452 (23.95%) | 229,851 (21.15%) |
| 35 to 49 | 10,069,444 (24.72%) | 273,973 (25.21%) |
| 50+ | 11,812,112 (28.99%) | 371,772 (34.21%) |
| **Elixhauser Comorbidity Index** | | |
| 0 | 38,294,865 (94%) | 969,962 (89.26%) |
| 1 | 2,354,975 (5.78%) | 112,819 (10.38%) |
| 2 | 69,213 (0.17%) | 2,905 (0.27%) |
| 3+ | 20,862 (0.05%) | 1,034 (0.1%) |
| **US Census Region of Residence** | | |
| Midwest | 10,056,251 (24.68%) | 265,029 (24.39%) |
| Northeast | 7,036,675 (17.27%) | 168,888 (15.54%) |
| South | 16,771,293 (41.17%) | 496,176 (45.66%) |
| West | 6,875,696 (16.88%) | 156,627 (14.41%) |
| **Urban / Rural Status of Residence** | | |
| Rural | 6,308,636 (15.49%) | 211,503 (19.46%) |
| Urban | 34,431,279 (84.51%) | 875,217 (80.54%) |
| **COVID-19 Hot-Spot of Residence** | | |
| Hotspot - No | 18,676,928 (45.84%) | 442,704 (40.74%) |
| Hotspot - Yes | 22,062,987 (54.16%) | 644,016 (59.26%) |
| **Area Deprivation Index (*by Quartile*)** | | |
| Quartile 1 *(Low Deprivation)* | 16,869,885 (41.41%) | 385,242 (35.45%) |
| Quartile 2 | 9,163,847 (22.49%) | 256,311 (23.59%) |
| Quartile 3 | 8,582,511 (21.07%) | 257,559 (23.7%) |
| Quartile 4 *(High Deprivation)* | 6,123,672 (15.03%) | 187,608 (17.26%) |
| **Internet Connectivity (*# of households with internet access per 1000 at zip code level*)** | | |
| 0-499 | 164,834 (0.4%) | 5,276 (0.49%) |
| 500-1000 | 40,575,081 (99.6%) | 1,081,444 (99.51%) |
| **Type of Insurance Plan in 2019** | | |

© 2022 Hatef E et al. *JAMA Network Open.*

| | | |
|---|---|---|
| High Deductible | 10,623,412 (26.08%) | 299,539 (27.56%) |
| HMO | 2,435,534 (5.98%) | 51,724 (4.76%) |
| Standard PPO | 27,680,969 (67.95%) | 735,457 (67.68%) |

© 2022 Hatef E et al. *JAMA Network Open.*

**eTable 8. In-Person and Telehealth Ambulatory Encounters for 40.7 million Continuously Enrolled Insured Persons During Post Implementation Phase (July-December); Comparing Pre COVID-19 (2019) and Post COVID-19 (2020) Periods**

| **Type of Service** | **Summer Plateau** (July-September) | | **Fall Surge** (October-December) | |
|---|---|---|---|---|
| | **2019-Pre COVID-19** | **2020-Post COVID-19** | **2019-Pre COVID-19** | **2020-Post COVID-19** |
| **Total Ambulatory Encounters Per Enrollee** | 1.20 | 1.20 | 1.26 | 1.25 |
| **In Person Ambulatory Encounters Per Enrollee** | 1.20 | 1.00 | 1.25 | 1.04 |
| **% of Total Encounters Via Telehealth** *(Distribution of telehealth modality below)* | 147,860 (0.36%) | 8,170,597 (20.06%) | 217,512 (0.53%) | 8,583,684 (21.07%) |
| • **Video Supported** | 54,631 (36.95%) | 6,547,014 (80.13%) | 71,685 (32.96%) | 6,803,596 (79.26%) |
| • **Telephone** | 85,080 (57.54%) | 543,849 (6.66%) | 134,751 (61.95%) | 559,614 (6.52%) |
| • **Other or Unspecified** | 8,149 (5.51%) | 1,079,734 (13.21%) | 11,076 (5.09%) | 1,220,474 (14.22%) |
| **% Enrollees with 1+ Ambulatory Encounter of Any Kind** | 18,072,540 (44.36%) | 17,448,169 (42.83%) | 18,675,255 (45.84%) | 17,894,218 (43.92%) |
| **% Enrollees with 1+ Telehealth Encounters** | 105,734 (0.26%) | 3,611,386 (8.86%) | 159,370 (0.39%) | 3,777,007 (9.27%) |

© 2022 Hatef E et al. *JAMA Network Open.*

**eTable 9. Ambulatory Encounters and Percentage Telehealth During 2019 and 2020: Unadjusted Results Broken Down by Characteristics of Persons and Their Residence Location**

| Patient-Level Characteristics | Patient, No (%) | Encounters per person, No. (% that were telehealth) | | | | | |
|---|---|---|---|---|---|---|---|
| | | Summer Plateau (July-September) | | | Fall Surge (October-December) | | |
| | | 2019-Pre COVID-19 | 2020-Post COVID-19 | | 2019-Pre COVID-19 | 2020-Post COVID-19 | |
| | | All Persons[a] | All Persons[a] | Persons with 1+ encounter | All Persons[a] | All Persons[a] | Persons with 1+ encounter |
| **Column Mean** | | **1.20** | **1.20** | **2.81 (16.7%)** | **1.26** | **1.25** | **2.84 (16.9%)** |
| **Age in 2019** | | | | | | | |
| 0 to 17 | 9,102,907 (22.34%) | 1.05 | 0.93 | 2.65 (18.93%) | 1.18 | 0.96 | 2.75 (19.51%) |
| 18 to 34 | 9,755,452 (23.95%) | 0.96 | 1.04 | 2.78 (25.01%) | 0.96 | 1.09 | 2.77 (25.52%) |
| 35 to 49 | 10,069,444 (24.72%) | 1.19 | 1.23 | 2.82 (19.02%) | 1.23 | 1.28 | 2.82 (19.07%) |
| 50+ | 11,812,112 (28.99%) | 1.53 | 1.52 | 2.89 (12.66%) | 1.58 | 1.56 | 2.93 (12.49%) |
| **Elixhauser Comorbidity Index** | | | | | | | |
| 0 | 38,294,865 (94%) | 1.10 | 1.13 | 2.75 (18.15%) | 1.15 | 1.17 | 2.78 (18.39%) |
| 1 | 2,354,975 (5.78%) | 2.72 | 2.33 | 3.28 (16.03%) | 2.85 | 2.34 | 3.28 (15.64%) |
| 2 | 69,213 (0.17%) | 5.00 | 3.62 | 4.61 (19.76%) | 5.43 | 3.46 | 4.44 (19.4%) |
| 3+ | 20,862 (0.05%) | 6.24 | 4.49 | 5.38 (19.08%) | 6.81 | 4.22 | 5.1 (18.27%) |
| **US Census Region of Residence** | | | | | | | |
| Midwest | 10,056,251 (24.68%) | 1.20 | 1.23 | 2.97 (15.64%) | 1.28 | 1.27 | 3.01 (16.94%) |
| Northeast | 7,036,675 (17.27%) | 1.28 | 1.28 | 2.87 (25.11%) | 1.32 | 1.33 | 2.91 (25.77%) |
| South | 16,771,293 (41.17%) | 1.18 | 1.21 | 2.64 (14.68%) | 1.24 | 1.25 | 2.67 (13.33%) |
| West | 6,875,696 (16.88%) | 1.16 | 1.07 | 2.97 (21.86%) | 1.20 | 1.10 | 2.98 (23.79%) |
| **Urban / Rural Status of Residence** | | | | | | | |
| Rural | 6,308,636 (15.49%) | 1.03 | 1.05 | 2.47 (10.23%) | 1.09 | 1.07 | 2.49 (10.99%) |
| Urban | 34,431,279 (84.51%) | 1.23 | 1.23 | 2.87 (19.13%) | 1.29 | 1.28 | 2.9 (19.19%) |
| **COVID-19 Hot-Spot of Residence** | | | | | | | |
| Hotspot - No | 24,304,495 (59.66%) | 1.25 | 1.25 | 2.93 (19.92%) | 1.30 | 1.31 | 2.93 (19.73%) |
| Hotspot - Yes | 16,435,420 (40.34%) | 1.13 | 1.13 | 2.58 (12.16%) | 1.14 | 1.07 | 2.63 (14.97%) |
| **Area Deprivation Index (*by Quartile*)** | | | | | | | |
| Quartile 1 *(Low)* | 16,869,885 (41.41%) | 1.34 | 1.32 | 3.04 (21.74%) | 1.40 | 1.38 | 3.08 (21.96%) |
| Quartile 2 | 9,163,847 (22.49%) | 1.15 | 1.14 | 2.71 (15.76%) | 1.20 | 1.18 | 2.73 (16.04%) |

© 2022 Hatef E et al. *JAMA Network Open.*

| | | | | | | | |
|---|---|---|---|---|---|---|---|
| Quartile 3 | 8,582,511 (21.07%) | 1.11 | 1.12 | 2.62 (14.01%) | 1.16 | 1.15 | 2.64 (14.21%) |
| Quartile 4 *(High)* | 6,123,672 (15.03%) | 1.04 | 1.08 | 2.56 (14.18%) | 1.08 | 1.10 | 2.57 (13.73%) |
| **Internet Connectivity *(# of households with internet access per 1000 at zip code level*)** | | | | | | | |
| 0-499 | 164,834 (0.4%) | 0.97 | 1.03 | 2.41 (2.23%, 8.63%) | 1.02 | 1.03 | 2.4 (2%, 7.63%) |
| 500-1000 | 40,575,081 (99.6%) | 1.20 | 1.20 | 2.81 (1.11%, 13.36%) | 1.26 | 1.25 | 2.84 (1.1%, 13.39%) |
| **Type of Insurance Plan in 2019** | | | | | | | |
| High Deductible | 10,623,412 (26.08%) | 1.04 | 1.05 | 2.56 (15.14%) | 1.09 | 1.10 | 2.59 (15.04%) |
| HMO | 2,435,534 (5.98%) | 1.21 | 1.06 | 2.93 (29.42%) | 1.22 | 1.09 | 2.96 (28.41%) |
| Standard PPO | 27,680,969 (67.95%) | 1.26 | 1.27 | 2.89 (17.97%) | 1.32 | 1.32 | 2.92 (18.33%) |

[a] The entire sample (of users and nonusers) continuously enrolled in commercial insurance plans from July 1, 2019, through December 31, 2020. These unadjusted rates are reported as the number of telehealth eligible ambulatory encounters per enrollee and the percentage of these encounters that took place via telehealth during each 3-month study period (July-September and October-December). All rows are calculated at the person level.
[b] Telephone% and video encounters%.

© 2022 Hatef E et al. *JAMA Network Open.*

**eTable 10. Ambulatory Encounters and Percentage Telehealth During 2019 and 2020: Unadjusted Encounter Level Results Broken Down by Characteristics of the Encounter**

| Encounter Level Characteristics | Encounters per person, No. (% that were telehealth) | | | | | |
|---|---|---|---|---|---|---|
| | Summer Plateau (July-September) | | | Fall Surge (October-December) | | |
| | 2019-Pre COVID-19 | 2020-Post COVID-19 | | 2019- Pre COVID | 2020-Post COVID-19 | |
| | All Persons[a] | All Persons[a] | Persons with 1+ encounter | All Persons[a] | All Persons[a] | Persons with 1+ encounter |
| **Column Mean for Entire Subgroup** | **1.20** | **1.20** | **2.81 (16.7%)** | **1.26** | **1.25** | **2.84 (16.9%)** |
| **Type of Ambulatory Encounter** | | | | | | |
| Evaluation and Management Office Encounter | 0.77 | 0.76 | 1.78 (14.64%) | 0.83 | 0.80 | 1.82 (14.59%) |
| Behavioral Health | 0.16 | 0.18 | 0.41 (52.15%) | 0.16 | 0.18 | 0.41 (54.66%) |
| Rehabilitation | 0.07 | 0.08 | 0.18 (10.02%) | 0.07 | 0.07 | 0.17 (10.81%) |
| Other | 0.19 | 0.18 | 0.43 (1.87%) | 0.20 | 0.19 | 0.44 (1.46%) |
| **Provider Specialty** | | | | | | |
| Primary Care MD/DO | 0.33 | 0.31 | 0.72 (15.03%) | 0.36 | 0.32 | 0.72 (15.42%) |
| Medical Specialist MD/DO | 0.11 | 0.11 | 0.27 (13.97%) | 0.11 | 0.11 | 0.26 (13.19%) |
| Surgical Specialist MD/DO | 0.10 | 0.10 | 0.23 (3.77%) | 0.10 | 0.10 | 0.22 (3.51%) |
| Behavioral Health MD/DO/Non-MD | 0.08 | 0.09 | 0.21 (56.38%) | 0.08 | 0.09 | 0.21 (59.73%) |
| Rehabilitation MD/DO/Non-MD | 0.16 | 0.16 | 0.36 (2.95%) | 0.17 | 0.16 | 0.36 (2.53%) |
| Other Physician | 0.25 | 0.27 | 0.64 (25.11%) | 0.27 | 0.29 | 0.66 (24.6%) |
| Physician Assistant /Nurse Practitioner | 0.09 | 0.10 | 0.23 (13.33%) | 0.10 | 0.11 | 0.25 (13.94%) |
| Other non-Physician | 0.06 | 0.06 | 0.14 (18.61%) | 0.06 | 0.07 | 0.15 (18.56%) |
| **Primary Diagnosis** | | | | | | |
| Diabetes | 0.03 | 0.03 | 0.06 (14.32%) | 0.03 | 0.03 | 0.06 (13.38%) |
| Hypertension | 0.03 | 0.03 | 0.08 (12.99%) | 0.03 | 0.03 | 0.08 (11.56%) |
| Behavioral Health | 0.23 | 0.25 | 0.58 (50.31%) | 0.23 | 0.25 | 0.57 (52.56%) |
| Cancer | 0.02 | 0.03 | 0.06 (8.2%) | 0.02 | 0.03 | 0.06 (7.93%) |
| Well Child Care | 0.01 | 0.01 | 0.03 (0.3%) | 0.01 | 0.01 | 0.02 (0.29%) |
| COVID-19 Diagnosis | NA | 0.01 | 0.01 (30.25%) | NA | 0.02 | 0.04 (26.17%) |
| Other Chronic Diagnosis | 0.22 | 0.21 | 0.5 (12.81%) | 0.22 | 0.21 | 0.48 (12.49%) |
| Other Acute Diagnosis | 0.66 | 0.64 | 1.49 (7.97%) | 0.71 | 0.68 | 1.54 (7.9%) |

© 2022 Hatef E et al. *JAMA Network Open.*

| | | | | | | |
|---|---|---|---|---|---|---|
| **Continuity of Patient Encounter/ Problem** | | | | | | |
| Existing Patient Encounters | 0.93 | 0.89 | 2.08 (17.99%) | 0.97 | 0.92 | 2.09 (18.15%) |
| New Patient Encounters | 0.27 | 0.31 | 0.72 (17.73%) | 0.29 | 0.33 | 0.75 (17.95%) |
| Existing Problem Encounters | 0.78 | 0.74 | 1.73 (18.7%) | 0.80 | 0.75 | 1.72 (18.81%) |
| New Problem Encounters | 0.42 | 0.46 | 1.08 (16.67%) | 0.46 | 0.49 | 1.12 (17.01%) |

[a] The full study sample included persons continuously enrolled from July 1, 2019, through December 31, 2020. These unadjusted rates are reported as the number of telehealth eligible ambulatory encounters per enrollee that took place during each 3-month study period (July-September and October-December). Percentages represent the proportion of encounters that took place via telehealth. Note that all rows were calculated at encounter level and that the “all persons” columns include all enrollees whether or not they used any services.

© 2022 Hatef E et al. *JAMA Network Open.*

**eTable 11. Summary of Acute and Chronic Ambulatory Care Sensitive Conditions and Subsequent Utilization Patterns in 2020**

| Condition | Total Members | Members with Any Follow-up Encounter | Members with ED Follow-up | Members with Hospitalization Follow-up |
|---|---|---|---|---|
| **ACUTE - Ambulatory Care Sensitive Conditions** | | | | |
| **Acute bronchitis** | | | | |
| First Encounter: Telehealth | 9,245 | 6,981 (75.51%) | 384 (4.15%) | 306 (3.31%) |
| First Encounter: In-Person | 30,381 | 20,948 (68.95%) | 1,038 (3.42%) | 903 (2.97%) |
| **Acute pharyngitis** | | | | |
| First Encounter: Telehealth | 36,399 | 24,933 (68.5%) | 690 (1.9%) | 503 (1.38%) |
| First Encounter: In-Person | 189,567 | 105,755 (55.79%) | 2,952 (1.56%) | 2,218 (1.17%) |
| **Acute pyelonephritis** | | | | |
| First Encounter: Telehealth | 389 | 318 (81.75%) | 28 (7.2%) | 20 (5.14%) |
| First Encounter: In-Person | 1,655 | 1,341 (81.03%) | 114 (6.89%) | 85 (5.14%) |
| **Acute tonsillitis** | | | | |
| First Encounter: Telehealth | 3,758 | 2,427 (64.58%) | 93 (2.47%) | 40 (1.06%) |
| First Encounter: In-Person | 23,424 | 14,177 (60.52%) | 659 (2.81%) | 252 (1.08%) |
| **Acute upper respiratory infections of multiple and unspecified sites** | | | | |
| First Encounter: Telehealth | 49,336 | 34,966 (70.87%) | 1,348 (2.73%) | 945 (1.92%) |
| First Encounter: In-Person | 180,034 | 101,290 (56.26%) | 3,872 (2.15%) | 2,661 (1.48%) |
| **Pneumonia, unspecified organism** | | | | |
| First Encounter: Telehealth | 1,402 | 1,286 (91.73%) | 82 (5.85%) | 69 (4.92%) |
| First Encounter: In-Person | 7,420 | 6,290 (84.77%) | 373 (5.03%) | 421 (5.67%) |
| **Convulsions, not elsewhere classified** | | | | |
| First Encounter: Telehealth | 1,022 | 675 (66.05%) | 32 (3.13%) | 44 (4.31%) |
| First Encounter: In-Person | 3,099 | 2,246 (72.48%) | 121 (3.9%) | 119 (3.84%) |
| **Diseases of pulp and periapical tissues** | | | | |
| First Encounter: Telehealth | 2,480 | 1,348 (54.35%) | 89 (3.59%) | 47 (1.9%) |
| First Encounter: In-Person | 10,570 | 5,519 (52.21%) | 362 (3.42%) | 209 (1.98%) |
| **Other diseases of lip and oral mucosa** | | | | |
| First Encounter: Telehealth | 2,271 | 1,543 (67.94%) | 50 (2.2%) | 35 (1.54%) |
| First Encounter: In-Person | 14,573 | 10,077 (69.15%) | 336 (2.31%) | 253 (1.74%) |
| **Stomatitis and related lesions** | | | | |
| First Encounter: Telehealth | 2,187 | 1,446 (66.12%) | 79 (3.61%) | 40 (1.83%) |
| First Encounter: In-Person | 12,284 | 7,928 (64.54%) | 350 (2.85%) | 246 (2%) |

© 2022 Hatef E et al. *JAMA Network Open.*

| CHRONIC - Ambulatory Care Sensitive Condition | | | | |
|---|---|---|---|---|
| **Asthma** | | | | |
| First Encounter: Telehealth | 23,878 | 15,231 (63.79%) | 533 (2.23%) | 398 (1.67%) |
| First Encounter: In-Person | 72,918 | 45,301 (62.13%) | 1,617 (2.22%) | 1,241 (1.7%) |
| **Epilepsy and recurrent seizures** | | | | |
| First Encounter: Telehealth | 3,013 | 1,692 (56.16%) | 66 (2.19%) | 68 (2.26%) |
| First Encounter: In-Person | 7,645 | 4,632 (60.59%) | 187 (2.45%) | 195 (2.55%) |
| **Essential (primary) hypertension** | | | | |
| First Encounter: Telehealth | 42,450 | 27,753 (65.38%) | 628 (1.48%) | 867 (2.04%) |
| First Encounter: In-Person | 215,227 | 144,220 (67.01%) | 3,262 (1.52%) | 4,395 (2.04%) |
| **Heart failure** | | | | |
| First Encounter: Telehealth | 556 | 456 (82.01%) | 21 (3.78%) | 51 (9.17%) |
| First Encounter: In-Person | 3,908 | 3,418 (87.46%) | 173 (4.43%) | 343 (8.78%) |
| **Hypertensive heart disease** | | | | |
| First Encounter: Telehealth | 1,259 | 948 (75.3%) | 35 (2.78%) | 68 (5.4%) |
| First Encounter: In-Person | 7,551 | 5,976 (79.14%) | 229 (3.03%) | 354 (4.69%) |
| **Iron deficiency anemia** | | | | |
| First Encounter: Telehealth | 2,812 | 2,440 (86.77%) | 100 (3.56%) | 130 (4.62%) |
| First Encounter: In-Person | 9,174 | 7,652 (83.41%) | 330 (3.6%) | 464 (5.06%) |
| **Other and unspecified noninfective gastroenteritis and colitis** | | | | |
| First Encounter: Telehealth | 7,586 | 5,698 (75.11%) | 333 (4.39%) | 212 (2.79%) |
| First Encounter: In-Person | 21,929 | 14,995 (68.38%) | 888 (4.05%) | 622 (2.84%) |
| **Other chronic obstructive pulmonary disease** | | | | |
| First Encounter: Telehealth | 1,961 | 1,471 (75.01%) | 74 (3.77%) | 94 (4.79%) |
| First Encounter: In-Person | 9,165 | 6,906 (75.35%) | 323 (3.52%) | 445 (4.86%) |
| **Type 1 diabetes mellitus** | | | | |
| First Encounter: Telehealth | 689 | 491 (71.26%) | 24 (3.48%) | 20 (2.9%) |
| First Encounter: In-Person | 4,365 | 3,395 (77.78%) | 139 (3.18%) | 180 (4.12%) |
| **Type 2 diabetes mellitus** | | | | |
| First Encounter: Telehealth | 8,798 | 6,839 (77.73%) | 115 (1.31%) | 227 (2.58%) |
| First Encounter: In-Person | 52,817 | 39,747 (75.25%) | 931 (1.76%) | 1,524 (2.89%) |
| **Unspecified Disorders** | | | | |
| First Encounter: Telehealth | 1,479 | 865 (58.49%) | 50 (3.38%) | 39 (2.64%) |
| First Encounter: In-Person | 6,044 | 3,443 (56.97%) | 226 (3.74%) | 135 (2.23%) |

All ambulatory care sensitive conditions (grouped by 3-digit ICD-10 categories) were selected based on sufficient prevalence within our study population and if there was evidence of telehealth utilization for the conditions. Enrollees that had encounters related to the condition in the 90-day period before the first encounter within the study period were removed from the cohort. Follow-up encounters were counted if there were any subsequent encounters to a provider within 14 days after the initial encounter.
ED: Emergency Department

© 2022 Hatef E et al. *JAMA Network Open.*

**eTable 12. Counts and Percentage of Initial Ambulatory Encounters for Chronic Ambulatory Care Sensitive Conditions by Severity Level and Encounter Type**

| Severity Level | In-Person | Telehealth | All Encounters |
|---|---|---|---|
| **1** | 378961 (92.26%) | 84583 (89.52%) | 463544 (91.75%) |
| **2** | 30452 (7.41%) | 9580 (10.14%) | 40032 (7.92%) |
| **3** | 1330 (0.32%) | 318 (0.34%) | 1648 (0.33%) |
| **All Levels** | 410743 (100%) | 94481 (100%) | 505224 (100) |

© 2022 Hatef E et al. *JAMA Network Open.*

## eFigure1: Sample Selection and Subgroup Identification Diagram

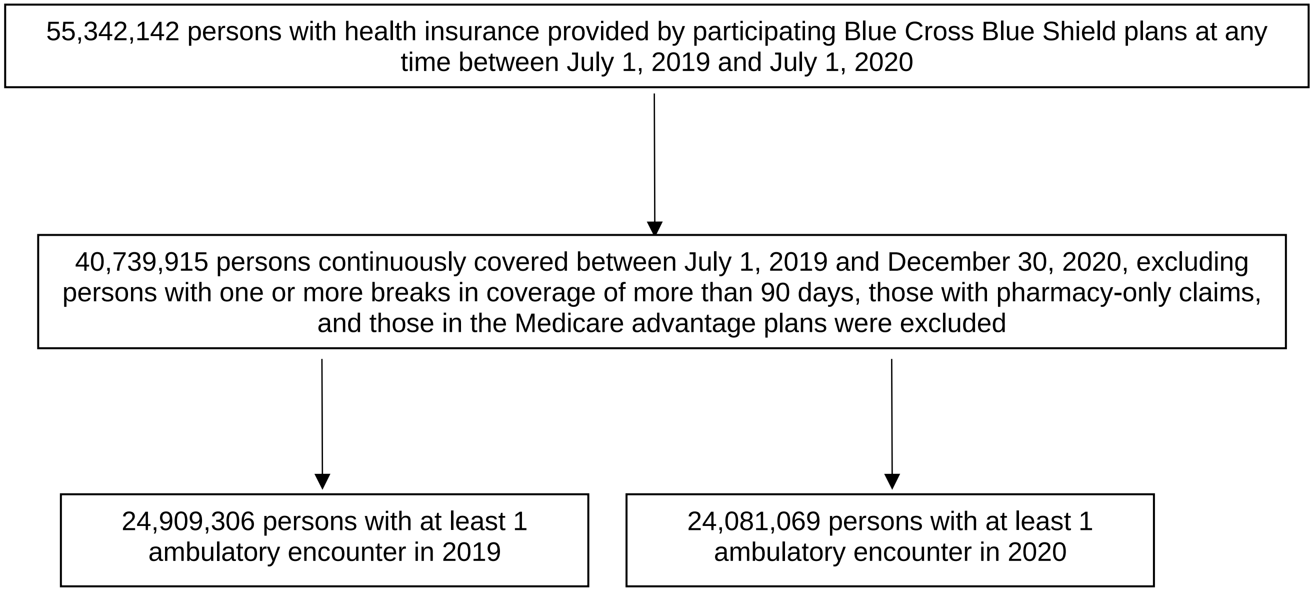


Ambulatory encounters are encounters for a set of CPT® and HCPCS codesthat in 2020 are eligible for payment on a remote, telehealth basis. See Table 1 for a listing of codes. Inpatient and hospital emergency department places of service are excluded from the definition, as are service codes that are explicitly defined as inpatient or emergency services. A encounter or encounter is defined as a unique combination of the member, rendering physician, date of service, and place of service.
Only 1.4% of the cohort had any gap in coverage and of these, the majority of those with a gap (66%) had a gap of one month or less.
CPT: Current Procedural Terminology, HCPC: Healthcare Common Procedure Coding.

© 2022 Hatef E et al. *JAMA Network Open.*

## eFigure 2. The ratio of 2020 to 2019 Ambulatory (AMB) Clinical Encounters by Week, July through December

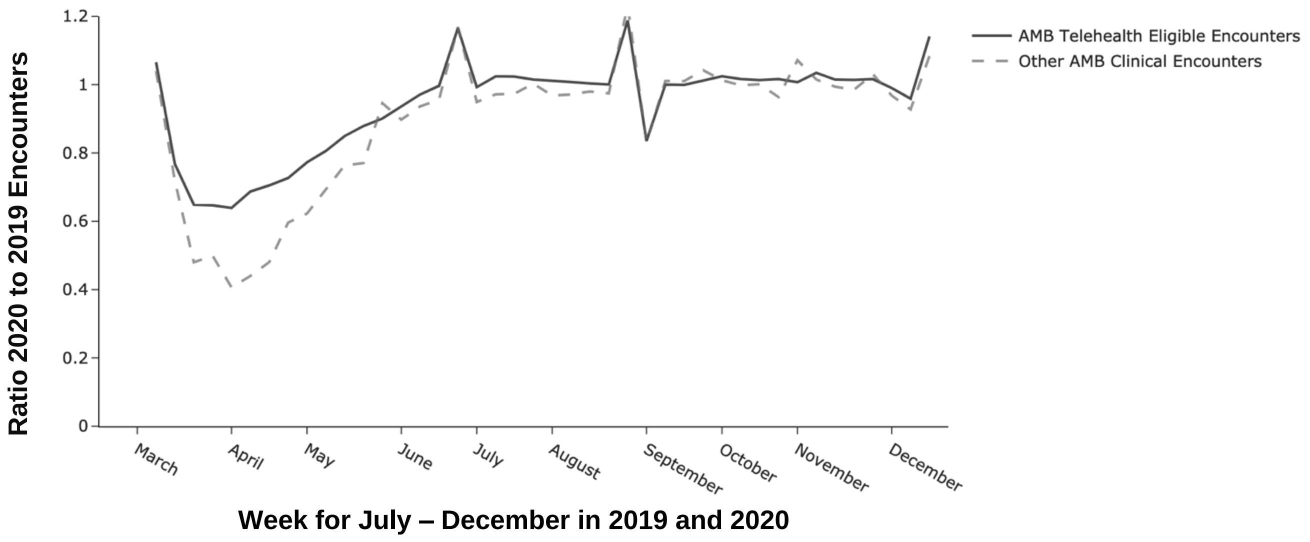


The graph distinguishes between *telehealth-eligible services* (our listof 264 codes) and all other services provided on an ambulatory basis, except for lab tests and ED encounters. Together these two categories correspond to almost all ambulatory care that requiresa clinical encounter.

By the second week of April 2020, Other ambulatory (AMB) encounters had decreased to 40.1% of 2019 levels. In contrast, telehealth eligible services were less severely impacted and decreased to just 64% of 2019 Levels. By the end of June, both categories have steadily climbed back almost to 2019 levels and remained consistent throughout the rest of 2020.

© 2022 Hatef E et al. *JAMA Network Open.*

## eFigure 3. Telehealth-eligible Ambulatory (AMB) Encounters per 1000 Enrollees by Week, March-June 2019 and 2020

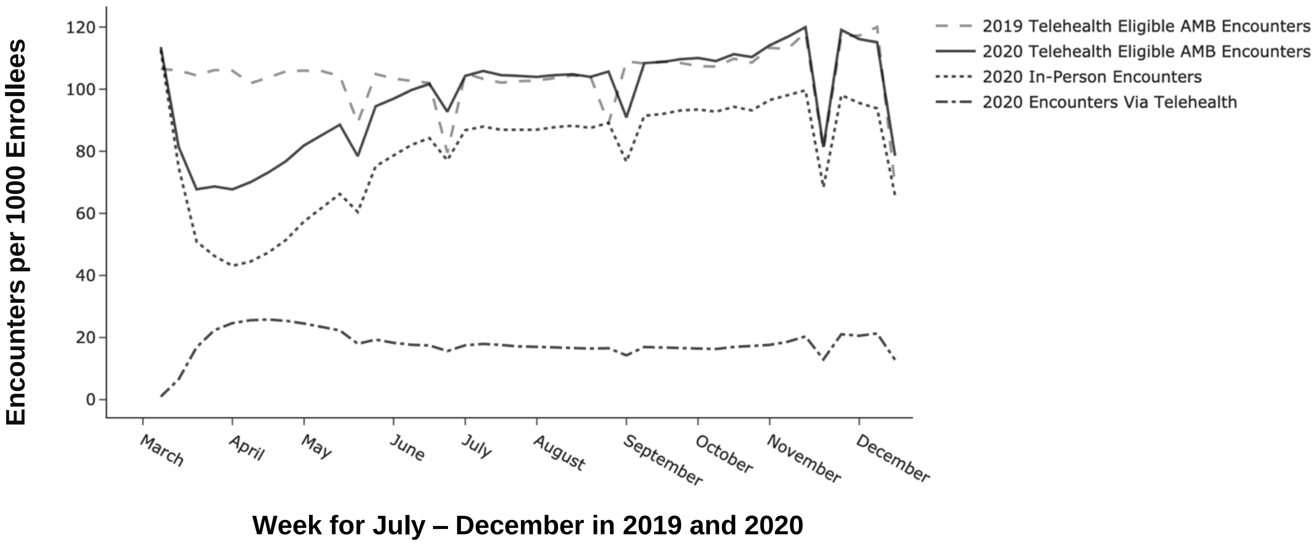


The graphs are based on encounters per 1000 enrollees and explain a disparity in impact. This graph focuses exclusively on *telehealth-eligible services* and distinguishes between those provided on an *in-person* basis and those provided via *telehealth*.

In-person encounters in 2020 were severely impacted and dropped to a low of 43.1 encounters per 1000, but this drop was partially offset by a sharp increase in telehealth - from less than 1 encounter per 1000 at the beginning of March, to a peak of 25.8 telehealth encounters per 1000 at the end of April. By the end of June, telehealth encounters decreased to a rate of 17.5 per 1000, and remained fairly consistent in this range throughout the rest of 2020. Telehealth played a critical role in ensuring continuity and availability of care at the peak of the crisis in 2020, and remained prevalent even after the peak of the crisis.
